# Benchmarking Self-Supervised Contrastive Learning Methods for Image-Based Plant Phenotyping

**DOI:** 10.34133/plantphenomics.0037

**Published:** 2023-04-03

**Authors:** Franklin C. Ogidi, Mark G. Eramian, Ian Stavness

**Affiliations:** Department of Computer Science, University of Saskatchewan, Saskatoon, Canada.

## Abstract

The rise of self-supervised learning (SSL) methods in recent years presents an opportunity to leverage unlabeled and domain-specific datasets generated by image-based plant phenotyping platforms to accelerate plant breeding programs. Despite the surge of research on SSL, there has been a scarcity of research exploring the applications of SSL to image-based plant phenotyping tasks, particularly detection and counting tasks. We address this gap by benchmarking the performance of 2 SSL methods—momentum contrast (MoCo) v2 and dense contrastive learning (DenseCL)—against the conventional supervised learning method when transferring learned representations to 4 downstream (target) image-based plant phenotyping tasks: wheat head detection, plant instance detection, wheat spikelet counting, and leaf counting. We studied the effects of the domain of the pretraining (source) dataset on the downstream performance and the influence of redundancy in the pretraining dataset on the quality of learned representations. We also analyzed the similarity of the internal representations learned via the different pretraining methods. We find that supervised pretraining generally outperforms self-supervised pretraining and show that MoCo v2 and DenseCL learn different high-level representations compared to the supervised method. We also find that using a diverse source dataset in the same domain as or a similar domain to the target dataset maximizes performance in the downstream task. Finally, our results show that SSL methods may be more sensitive to redundancy in the pretraining dataset than the supervised pretraining method. We hope that this benchmark/evaluation study will guide practitioners in developing better SSL methods for image-based plant phenotyping.

## Introduction

Phenotyping is an essential part of a crop breeding program, where the plant breeder is tasked with crossing parents and selecting progeny with desirable and improved traits, including, among other things, yield and resistance to biotic and abiotic stress [1–3]. Phenotyping also constitutes a major bottleneck in crop breeding programs due to the substantial number of progenies involved and the need to not miss a superior progeny in the selection process, which is typically labor-intensive [1,4]. Due to rapidly changing climate conditions and the growing global human population, there is a need to accelerate the crop breeding process to breed more resilient crops to variable environmental conditions while improving crop yield to ensure global food security [5].

In recent years, image-based plant phenotyping has emerged as a promising tool for alleviating the phenotyping bottleneck. Image-based plant phenotyping involves using one or more imaging technologies to capture images of plants over a growing season and applying image analysis tools to measure/extract plant traits in a nondestructive and (semi-) automated way [5]. This technique facilitates the high-throughput measurement of plant phenotypes in a short time frame. Compared to traditional and manual plant phenotyping methods, it offers the benefits of high precision in measurement and less cost in terms of human labor and time [6]. The main challenge for image-based plant phenotyping is to extract complex traits from the large amounts of data generated by high-throughput phenotyping platforms [5,7].

Deep learning methods are particularly well suited for simultaneously extracting semantically meaningful features from data and using those features to perform a set of tasks [8,9]. From raw data, they compose simpler patterns into more complex ones through multilayered computational models like convolutional neural networks. Deep learning has been a widely successful method for image-based tasks like image classification, object detection, and semantic segmentation [8]. Its application has also extended to image-based plant phenotyping tasks like plant classification [10–12], plant stress classification [13], plant detection [14], plant disease detection [15], and plant organ detection/counting [16,17]. Currently, most deep learning implementations follow the supervised learning paradigm, which requires labeled datasets that can be expensive and time-consuming to produce. However, it is possible to circumvent the need for large, annotated datasets by transferring knowledge from a source domain (dataset) or task to a target domain or task in a process known as transfer learning [18,19].

Transfer learning typically works by training a model on a source task and using the parameters of the trained model to either construct a fixed feature extractor or to initialize the parameters of a new model for the target task, which are then fine-tuned (updated during training). Transfer learning is a popular technique for image-based tasks, catalyzed by the ubiquity of models pretrained on large-scale benchmark datasets like ImageNet [20] and COCO [21]. In the domain of plant phenotyping, transfer learning has been used for tasks like weed classification [12], plant detection [14], and plant center localization [22]. While transfer learning works even when the domain of the source and target tasks are different, a study of over 2,000 transfer learning experiments shows that within-domain transfer maximizes positive transfer gains across many tasks, even when the domain of the source task is much broader than the target [18]. The transfer learning workflow for plant phenotyping tasks largely entails a source task that is trained in a supervised manner on a large, annotated dataset, which is often outside the domain of the target task. This workflow is not ideal for plant phenotyping, where the availability of large, annotated datasets is limited.

Self-supervised learning (SSL) methods provide a means to tackle the problem of enabling within-domain transfer while reducing the need for labeled data. SSL methods combine aspects of supervised and unsupervised learning. While they do not rely on explicit input–target pairs, they generate a supervisory signal or pseudo-label from the input data, which, in addition to an appropriate objective function, is used to solve a pretext task. The pretext task is typically only designed for learning useful features/representations of the data, which can be used to initialize the network weights for a downstream task of real-world importance. Among successful SSL approaches are contrastive learning methods like SimCLR [23] and momentum contrast (MoCo) v1/v2/v3 [24–26]; non-contrastive methods like BYOL [27], SimSiam [28], and Barlow Twins [29]; and clustering methods like SwAV [30] and SeLa [31]. These methods generate image-level features that may be unsuitable for dense prediction tasks like object detection and semantic segmentation, as they may discard important spatial information. This problem has prompted the development of dense SSL methods such as dense contrastive learning (DenseCL) [32], SetSim [33], PixPro [34], and ReSim [35]. Dense SSL methods promote exploiting local features that preserve spatial information and are more appropriate for dense prediction tasks.

Despite the rapid progress in the development of SSL methods, there has been relatively little research applying or evaluating SSL methods on image-based plant phenotyping tasks, especially detection and counting tasks. Detection and counting tasks are among the most impactful in plant phenotyping, as the number of plants/plant organs is an important yield component. It is also costly and time-consuming to perform these tasks manually for a large number of plants, let alone generate a large-scale annotated dataset for supervised learning applications.

In this work, we benchmark the transferability of contrastive SSL methods against the conventional supervised pretraining method on 4 downstream image-based plant phenotyping tasks: wheat head detection, plant instance detection, wheat spikelet counting, and leaf counting. Since these tasks involve local prediction, we explore the benefits of using a dense SSL approach (DenseCL [32]) over one that learns global representations (MoCo v2 [25]). We further investigate how the source domain affects transfer performance by comparing SSL results using 4 different source domain datasets: ImageNet [20], iNaturalist 2021 [36], the Plants subset of the iNaturalist 2021 dataset, and the TerraByte Field Crop (TFC) dataset [37]. These source domains were chosen to be successively closer to the target domain, starting from general images (ImageNet), to natural images (iNaturalist), to general plant images (plant images within iNaturalist), and finally to crop images (TFC). This setup allows us to assess whether general or more specialized pretraining datasets are more useful for image-based plant phenotyping. Data redundancy is also important for plant phenotyping datasets because commonly used imaging platforms, such as drones, tractors, and carts, capture images with substantial spatial overlap. Therefore, with the crop dataset, we study the effects of data redundancy on the quality of representations. Finally, we analyze the similarity of the internal representations learned by neural networks trained with the different representation learning methods.

Through a large set of SSL experiments, we report a number of novel findings. (a) Compared to MoCo v2 and DenseCL, supervised pretraining yields the best-performing model on all the downstream tasks, except the leaf counting task. (b) In most cases, a domain-specific but diverse pretraining dataset results in the best downstream performance across the different pretraining methods. (c) Compared to the supervised method, the SSL methods show more sensitivity to redundancy in the pretraining dataset. (d) There is a high degree of similarity in the internal representations of models trained with MoCo v2 versus DenseCL across all layers. In contrast, the internal representations of supervised versus self-supervised models start out similar in the early layers and quickly become more dissimilar in the last few layers. This study shows the promise of SSL for plant phenotyping in terms of leveraging unlabeled datasets, but also points to possible areas of improvement.

## Related Work

### Self-supervised representation learning

SSL is a form of representation learning that works with unlabeled data, usually by leveraging some aspect of the data such as distorting it and learning representations that are invariant to the distortions. Most SSL frameworks involve a 2-stage process. In the first stage (the pretraining stage), a model learns representations by solving a pretext task that does not require explicit manual supervision. The second stage involves using the pretrained model as a feature extractor or fine-tuning the model on a downstream task with supervised or unsupervised learning. Based on the idea that a good representation is linearly separable in some latent space [9], the quality of the representations learned in the pretraining stage is commonly evaluated using the linear evaluation protocol, in which a linear classifier is trained on the frozen representations.

Several pretext tasks have been explored in the literature. Some early pretext tasks like Exemplar-CNN [38] or predicting image rotations [39] are based on the idea that applying some transformations to an image does not change its semantic meaning. Therefore, a deep learning network can learn semantically meaningful representations from the data without requiring explicit labels by learning to be invariant to the transformations. Other works have explored pretext tasks based on the spatial relationship between image patches [40–42], generative modeling by reconstructing distorted image inputs [43–45], and estimating and maximizing the mutual information between different views of images [46,47]. In recent years, the instance discrimination task has been among the most successful SSL pretext tasks for image-based tasks. The instance discrimination task treats an image as a distinct category and learns to discriminate between individual instances without considering broader semantic categories and usually with the help of a contrastive loss function [48].

Contrastive learning methods, through a contrastive loss, learn to pull positive samples closer in an embedding space while simultaneously pushing negative samples apart. Positive samples are considered different (augmented) views of a given image for the instance discrimination task, while negative samples are other images in the dataset. SimCLR [23] and MoCo v2 [25] are 2 of the most popular contrastive SSL frameworks. They both include a relatively heavy data augmentation pipeline, a Siamese encoder with shared weights, and a nonlinear projection head, all optimized with the InfoNCE loss function [49]. They differ mainly in their approach to negative sampling. While SimCLR relies on a large batch size to get negative samples, the MoCo framework [24] uses a queue of feature vectors constructed on the fly via a momentum encoder. The approach of MoCo v2 improves efficiency and performance in terms of batch size, memory, training speed, and ImageNet linear classifier accuracy [25].

Many popular SSL frameworks, including SimCLR [23] and MoCo v2 [25], use global, image-level features for discriminative modeling, which may not sufficiently account for region-level or pixel-level details. Dense SSL methods have been developed to account for local features. The DenseCL framework [32] extends MoCo v2 for dense prediction tasks. It conducts local contrastive learning using pixel-level feature similarity to determine positive samples. Other dense SSL methods like ReSim [35] and DetCo [50] also extend MoCo v2 but use region-level similarity. However, they have comparable performance with DenseCL on popular object detection, semantic segmentation, and instance segmentation benchmarks.

### Prior benchmarking and evaluation studies

The area of research in evaluating self-supervised representation learning methods is motivated by the observation that most new SSL methods are developed and evaluated under a limited set of conditions. For instance, ImageNet [20] is the most commonly used dataset for pretraining, and evaluation on downstream tasks uses a small set of standard datasets. While this approach facilitates comparison between different methods, several studies have shown that ImageNet pretraining does not always have a positive transfer gain for most downstream tasks [18,36,51,52]. Motivated by these findings, prior works have tried to benchmark methods on different domains [36,53] and study the effects of dataset quantity [36,54], dataset quality [54], dataset domain [36,51,54], and task granularity [54] on downstream performance. Our work focuses on the agricultural domain and studies 3 important dimensions of SSL-based pretraining: (a) the effects of the pretraining algorithm on the downstream performance, (b) the effects of the pretraining dataset domain on the downstream performance, and (c) the effects of data redundancy on the quality of learned representations.

Our study is closely related to prior SSL benchmarking studies, such as Kotar et al. [51], which introduced a visual representation benchmark suite consisting of 4 pretraining algorithms, 4 pretraining datasets, and 20 downstream tasks [51]. The pretraining algorithms include the standard supervised learning framework and 3 self-supervised contrastive learning methods: MoCo [24], SwAV [30], and Pretext-Invariant Representation Learning (PIRL) [55]. The pretraining datasets include ImageNet [20], Places365 [56], Kinetics400 [57], and Taskonomy [58]. The downstream tasks cover various visual tasks with general images, including image classification, semantic segmentation, ego-motion estimation, and depth estimation. In contrast, our work focuses on the agricultural domain, and we evaluate different algorithms, datasets, and downstream tasks.

### SSL on agricultural images

There has been a paucity of research on applying SSL to agricultural images, especially for detection and counting tasks. There has been some research on a self-supervised clustering method for identifying leaf diseases [59] and a self-supervised segmentation method for segmenting leaves [60]. Marin Zapata et al. [61] refined ImageNet-pretrained representations on images of *Arabidopsis thaliana* by using a self-supervised approach that takes advantage of the similarity of plant images taken at relatively short, consecutive intervals. They demonstrated the applicability of the refined representations for phenotype clustering, mode of action discrimination (a classification task), and phenotype matching/retrieval. Güldenring and Nalpantidis [62] investigated a clustering-based self-supervised representation learning method for plant classification and segmentation tasks. They showed that self-supervised pretraining outperforms supervised pretraining on classification tasks but underperforms on segmentation tasks. This finding motivates the need to explore dense SSL methods for plant phenotyping tasks.

## Materials and Methods

### Experimental design

The objective of this work was to study how well representations learned in a self-supervised manner transfer to detection and counting tasks in the context of image-based plant phenotyping compared to representations learned in the standard fully supervised manner. We focused on 4 image-based plant phenotyping tasks: wheat head detection, plant instance detection, wheat spikelet counting, and leaf counting. These tasks are underexplored in the literature in the context of self-supervised representation learning. Since these tasks involve local predictions, we aimed to study the comparative performance between pretraining with an SSL method that optimizes image-level (global) features (MoCo v2 [25]) and one that optimizes for pixel-level (local) features (DenseCL [32]). We evaluated the performance on the downstream tasks using representations from 4 pretraining datasets: ImageNet [20], iNaturalist 2021 [36], the Plants supercategory of iNaturalist 2021, and the TFC dataset [37]. By using this diverse range of pretraining datasets, we aimed to explore the effects of the image domain of the datasets on the downstream tasks. Furthermore, by creating sub-datasets of the TFC dataset with varying degrees of redundancy, we aimed to study the effects of the degree of redundancy in the pretraining dataset on the quality of representations and transfer performance.

**Fig. 1.**
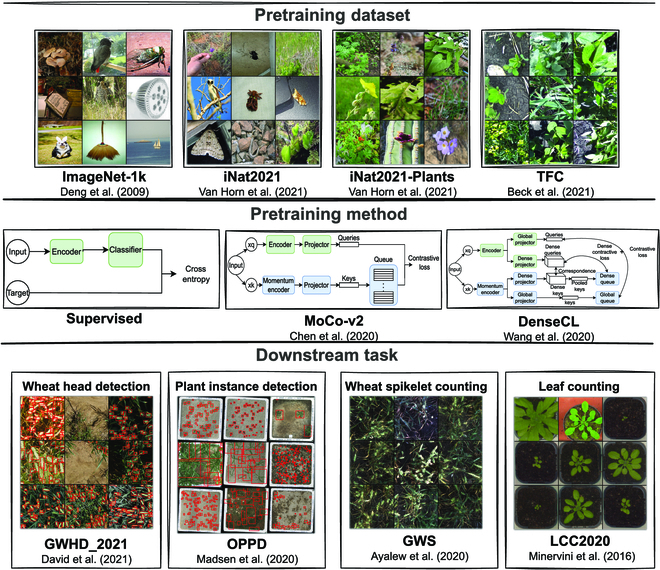
An overview of the major components of the experimental design. (Top–bottom) The pretraining datasets with domains ranging from general-purpose concepts to field crop images, similar to those seen in the downstream tasks; the pretraining methods; and the downstream tasks, including object detection tasks (with bounding boxes shown in red) and counting tasks. We train an encoder with the pretraining datasets for each pretraining method and fine-tune the weights on each downstream task.

We designed a set of experiments to achieve these objectives using the factorial experiment design method. The factorial experiment design method is a statistical experimental design method that allows the study of the effects of multiple factors on the performance of a system. We use it in this case to study the effects of the pretraining dataset and pretraining method on the downstream performance. As illustrated in Fig. 1, the major components of the experiment are the pretraining dataset, the pretraining method/algorithm, and the downstream task. Our workflow involved a pretraining step and a fine-tuning step. The models used in both steps share the same encoder. At the fine-tuning step, we initialized the weights of the encoder network with the final weights obtained from the pretraining step. We obtained pretrained weights for each combination of the pretraining dataset and pretraining method to initialize the encoder for each downstream task. We applied a representation similarity analysis technique to quantitatively compare the pretrained models’ internal representations. For the study on the effects of data redundancy, we used the linear evaluation protocol to evaluate the quality of the SSL representations. To highlight the effects of the pretraining step, we include the performance of the downstream task when trained from random initialization.

#### Pretraining datasets

The image domain plays an important role in the downstream performance when transferring knowledge between a source task and a target task [18,54]. To study the effects of the domain of the pretraining dataset on the transfer performance, we selected 4 large-scale datasets: ImageNet-1k [20], iNaturalist 2021 [36], iNaturalist 2021 Plants subset [36], and the TFC dataset [36]. These datasets were chosen with image domains that are successively closer to the image domain of the downstream task and include general-purpose concepts (ImageNet), natural world images (iNat2021), plant images (iNat2021 Plants), and field crop images (TFC). A comparison of domain distance between the pretraining and downstream datasets is shown in Fig. S4.

**ImageNet-1k.** ImageNet [20] has been the de facto standard for benchmarking representation learning algorithms since its introduction at the ImageNet Large Scale Visual Recognition Challenge, which was one of the catalysts for the deep learning revolution. While the entire ImageNet dataset currently contains about 14 million images covering approximately 22,000 categories, we used the most popular subset with 1.2 million training images representing 1,000 general-purpose concepts. Due to its ubiquity in benchmarking representation learning methods, we used it as a baseline to compare with the other datasets. Its image domain is also the farthest from the image domain of the downstream tasks.

**iNat2021 and iNat2021-plants.** The iNaturalist 2021 dataset [36] contains 2.7 million training images, 100,000 validation images, and 500,000 test images covering 10,000 species across the tree of life. We sampled a subset of images in the super category of Plants, which we call the iNat2021-Plants dataset. This subset contains 1.1 million training images, representing 4,271 species of plants. Both datasets represent a step closer to the image domain of the downstream tasks because they both contain plant images.

**The TFC dataset.** The TFC dataset [37] is a collection of field images of 5 common crops grown in Canada: soybean, faba bean, wheat, oats, and canola. The images are frames extracted from videos from a camera mounted on a tractor driven through the field. The fields were imaged periodically over the 2020 growing season and the middle part of the 2019 growing season (June-July), resulting in a total of about 542,000 video frames.

The dataset is largely uncurated, however. It has a high degree of similarity between consecutive frames extracted from the videos. It also contains images of field areas with little or no vegetation, which may have little value in the representation learning process. We leverage these data quality issues to study the influence of redundancy (in terms of the spatial overlap between extracted frames) in the pretraining dataset on the quality of representations and the downstream performance.

We created 5 sub-datasets from the original dataset with varying degrees of redundancy, as summarized in Table 1. First, we used only the images from the 2020 growing season as they contain metadata that allowed us to estimate the overlap between frames. We then removed the frames with little or no vegetation using the Excess Green minus Excess Red vegetation index [63]. These steps resulted in a dataset with 376,851 frames, representing the first sub-dataset TFC_1. We split this dataset into 302,585 training images, 33,417 validation images, and 40,849 test images using the stratified random sampling method. To minimize the computational burden of handling a large batch of high-resolution images during training, we sliced each frame in the training set into eight 640 × 640 nonoverlapping patches in a 2 × 4 grid, after upsampling them to a resolution of 2,560 × 1,440 using bilinear interpolation. We also removed patches with little to no vegetation. We constructed the second sub-dataset, TFC_2, from TFC_1 by selecting frames such that there is roughly a 75% spatial overlap between consecutive frames. The amount of frame overlap was determined using a heuristic that considers the tractor’s travel speed, the camera’s frame rate, and the ground sampling distance. Similarly, we constructed TFC_3, TFC_4, and TFC_5 subsets so that there is 50%, 25%, and no spatial overlap between consecutive frames for the area imaged, respectively. See Section A.1 in the Supplementary Materials for more details.

**Table 1.** The number of samples in each subset of the TFC dataset. The TFC_2, TFC_3, TFC_4, and TFC_5 subsets were sampled from the TFC_1 subset such that consecutive frames have roughly 75%, 50%, 25%, and no spatial overlap, respectively. Each frame in the training set was sliced into 8 patches of size 640 × 640, while the images in the validation and test sets are at full resolution. The TFC_1 subset contains the most redundancy while the TFC_5 subset contains the least redundancy.

Common name	Scientific name	Training					Validation	Test
		TFC_1	TFC_2	TFC_3	TFC_4	TFC_5		
Canola	*Brassica napus*	630,880	189,237	107,568	73,569	56,104	11,337	13,856
Faba bean	*Vicia faba*	77,594	22,109	12,774	8,260	6,586	1,806	2,208
Oat	*Avena sativa*	57,367	19,344	11,380	7,393	5,946	1,313	1,604
Soybean	*Glycine max*	866,822	263,529	146,552	102,780	78,329	17,496	21,390
Wheat	*Triticum aestivum*	64,306	21,296	12,641	8,538	6,662	1,465	1,791
**Total**		**1,696,889**	**515,515**	**290,915**	**200,540**	**153,627**	**33,417**	**40,849**

#### Pretraining methods

We considered 2 self-supervised contrastive representation learning methods based on the MoCo framework and compared their performance to the standard supervised representation learning method.

**Momentum contrast.** The MoCo [24] framework is based on the view of contrastive learning as a dictionary look-up problem. In this view, an encoded query is matched against a dictionary of encoded keys such that the query is similar to its matching key and dissimilar to others. MoCo is a mechanism for building large, consistent dictionaries to support unsupervised representation learning via a contrastive loss.

MoCo works by first generating different views of a batch of images through a stochastic augmentation pipeline. One batch of views is passed through a neural network to generate a batch of L2-normalized query representations *q*. The other batch of views is passed through a momentum-updated version of the first neural network to update a dictionary of keys {*k*_0_, *k*_1_, *k*_2_, …}. The key representations are also L2-normalized, and the dictionary is randomly initialized. The dictionary is kept large and decoupled from the mini-batch size by maintaining a queue of representations. The current mini-batch is enqueued at each training step, and the oldest are dequeued. The representations in the dictionary are kept consistent by using a momentum-based moving average of the query encoder to encode the keys. The MoCo framework learns useful representations by encouraging the encoders to produce representations that are similar for 2 views of the same image but dissimilar to other representations in the dictionary. This learning objective is formulated as minimizing the InfoNCE loss function [49], defined as follows:

$$L_{q}=-\text{log}\frac{\exp\left(q\cdot k_{+}/\tau \right)}{\sum_{i=0}^{K}‍\exp\left(q\cdot k_{i}/\tau \right)}$$ (1)

where *k*_+_ is the key in the dictionary matching the query *q* and *τ* is a temperature hyperparameter that controls the local separation and global uniformity of the embedding space [64].

In this work, we used MoCo v2 [25], which improves the original framework by increasing the strength of the augmentation pipeline and adding a nonlinear projection head after the encoders (see Fig. S1 for a conceptual illustration of the framework). Both these design features were introduced in SimCLR [23]. However, adopting them in the MoCo framework results in superior performance [25].

**Dense contras tive learning.** The development of DenseCL [32] is motivated by the need to improve unsupervised representations for dense prediction tasks like semantic segmentation and object detection. DenseCL builds on MoCo v2 by adding a dense projection head in parallel with the multilayer perceptron (MLP) projection head to optimize for the pairwise contrastive similarity between pixel-level features of different views of an image. Figure S2 illustrates the DenseCL architecture.

In the DenseCL framework, the dense projection head takes the dense feature maps before the global average pooling layer of the encoder as input. The dense projection head consists of identical 1 × 1 convolutional layers arranged similarly and with the same number of parameters as the MLP projection head. The output of the dense projection head is an *S_h_* × *S_w_* dense feature map, where *S_h_* and *S_w_* are the height and width of the dense feature map, respectively. For a given query *r*, representing a pixel in the dense feature map or a descriptor for a local part of the input image, and a set of encoded keys {*t*_0_, *t*_1_, *t*_2_, …}, DenseCL finds the positive keys by first establishing a dense correspondence between the backbone feature maps of the different views by matching each feature vector in one view with the most similar feature vector in the other view using the cosine similarity measure. The indices of the most similar feature vectors from the matching process are used to extract the positive keys *t*_+_ from the projected dense feature maps. The negative keys *t*_−_ are derived from the global average pooled vector of the projected dense feature map. Then, the dense contrastive loss is applied to pull the positive keys close together while pushing away the other negative keys:

$$L_{q}=-\;\text{log}\frac{\text{exp}(q\cdot k_{+}/\tau )}{\sum_{i=0}^{K}\text{exp}(q\cdot k_{i}/\tau )}$$ (2)

where *r^s^* is the *s*th feature vector from the *S_h_* × *S_w_* encoded queries.

The overall objective for DenseCL combines Eqs. 1 and 2 in the following way:

$$L=\left(1-\lambda \right)L_{q}+\lambda L_{r}$$ (3)

where the *λ* term balances the contribution of image-level features and local features to the optimization. By default, the *λ* term is 0.5.

**Supervised learning.** Supervised learning is the most widely used form of machine learning. It involves learning features from labeled data. The labels function as a supervisory signal because they allow the model to measure the difference between its output and the expected output, which is used as feedback to adapt the model’s parameters in order to improve its output. The canonical supervised learning pipeline is illustrated in Fig. S3.

We solved a supervised image classification task using the pretraining datasets and transferred the weights to the downstream tasks. We used the A2 training recipe proposed by Wightman et al. [65] for supervised training of ResNet [66] encoders on the ImageNet [20] dataset. Although the training recipe was developed with the ImageNet dataset, we applied it to all pretraining datasets used in this study. We used the timm library [67] to train the models.

#### Downstream tasks

The downstream tasks in this study can be grouped into 2 broad categories based on the type of annotation they use: object detection and object counting. The tasks in the object detection category (wheat head detection and plant instance detection) have bounding box annotations for the objects of interest (wheat heads and plants). On the other hand, the object counting tasks (leaf counting and wheat spikelet counting) use dot/keypoint annotations for the objects of interest (leaves and wheat spikelets). The datasets for these tasks have a relatively small number of samples, partly due to the difficulty in obtaining the annotations, making them suitable candidates for applying transfer learning. We investigate the effects of the representation learning method and the different characteristics of the pretraining dataset when transferring learned representations to these tasks.

In the following sections, we describe these tasks, their associated datasets, and implementation details.

**Wheat head detection.** The wheat head detection task poses the problem of localizing all the wheat heads in a given image. The accurate localization of wheat heads in the field can help better estimate wheat head density, which is an important yield component [68].

For this task, we used the 2021 Global Wheat Head Detection (GWHD_2021) dataset [69]. The GWHD_2021 dataset contains ∼6,500 1,024 × 1,024 RGB images and more than 275,000 annotated wheat heads. The images are sourced from 12 different countries across 5 continents and cover a variety of genotypes, environmental conditions, and developmental stages. The challenge is to solve the task while being robust to these variations. The GWHD_2021 dataset contains 47 sub-datasets/domains defined mainly by the location, date, and platform from which the images were acquired. The training set includes 3,657 images from Europe and Canada, while the validation and test sets contain 1,476 and 1,382 images, respectively, from North America (excluding Canada), Asia, Africa, and Oceania. This setup encourages solutions that are robust to domain shift, which occurs when the distribution of the data in the target domain (validation and test sets) is different from the distribution of the data in the source domain (training set) [70].

For this task, we used a Faster-RCNN [71] detector with a ResNet-50 [66] backbone and a feature pyramid network (FPN) [72]. Concretely, we used the PyTorch [73] implementation of the model (https://pytorch.org/vision/0.10/_modules/torchvision/models/detection/faster_rcnn.html#fasterrcnn_resnet50_fpn), which freezes the first 2 layers and all the batch normalization (BN) layers of the backbone network during fine-tuning. The idea of freezing the first 2 layers is based on the work of Fast-RCNN [74], which showed that the early layers learn generic features and are task-independent, so that fine-tuning or freezing them has no meaningful impact on the detection results, but freezing them improves training speed. We applied only the random horizontal flip augmentation during training and no augmentation during testing. To evaluate performance on this task, we used the average domain accuracy (ADA) [68], defined as follows:

$$\mathrm{ADA}=\frac{1}{D}\sum_{d=1}^{D}\frac{1}{n_{d}}*\sum_{i=1}^{n_{d}}{\mathrm{Acc}}_{d}(i)$$ (4)

where *D* is the number of domains/subdatasets, *n_d_* is the number of images in a domain, and *Acc_d_*(*i*) [68] is the detection accuracy for image *i* in domain *d*, computed as follows:

$$\mathrm{Ac}c_{d}\left(i\right)=\frac{\mathrm{TP}}{\mathrm{TP}+\mathrm{FP}+\mathrm{FN}}$$ (5)

where *TP* is the number of ground truth boxes that match one predicted box, *FP* is the number of predicted boxes with no ground truth match, and *FN* is the number of ground truth boxes with no matches. Two boxes match if their intersection over union (IoU) is greater than 0.5.

We trained the model using stochastic gradient descent (SGD) for ∼22k steps with a batch size of 8, a weight decay of 10^−4^, and a momentum of 0.9. We tuned the base learning rate on the validation set by performing a hyperparameter sweep across 5 logarithmically spaced values between 8 × 10^−2^ and 10^−3^. We applied a linear warmup for 500 steps from an initial learning rate, which we set by multiplying the base learning rate by 10^−3^. The learning rate was multiplied by 0.1 at the 14,592nd and 20,064th steps. We trained the model with mixed precision and reported the average on the test set after training with 5 different random seeds.

**Plant ins tance detection.** The plant instance detection task aims to localize all plant instances in a given image. Here, we used the Open Plant Phenotyping Database (OPPD) [14], which was introduced to support developing and comparing different plant detection and classification algorithms. The dataset contains 7,590 high-resolution RGB images with over 315,041 annotated plant objects representing 64,292 individual plants from 47 plant species, including 46 weed species commonly found in Denmark and some negative samples. The data collection process consisted of growing the plants in polystyrene boxes under 3 different growth conditions induced by varying nutrient and soil moisture levels and tracking them over 4 trial seasons. This process was designed to achieve high intra-species variability to help build more robust deep learning models, which, when deployed, can help build detailed plant population maps that support crop management decisions [14].

We used the same model architecture and implementation for this task as the wheat head detection task. Following previous work [14], we split the data using the *k*-fold cross-validation scheme, although we used *k* = 5 instead of *k* = 10 due to time and computational constraints. We split the data so that all images of a polystyrene box in a specific trial only appear in a single split, and boxes containing the same species are assigned to different splits. This method of splitting ensures minimal overlap between the training and validation sets [14]. We also cropped out the black background in the images during training and validation. This preprocessing step has been shown to improve detection performance [75]. After cropping, we resized the images to a resolution of 1,024 × 1,024 using bilinear interpolation and applied random horizontal flipping to the training images. We applied all augmentations at runtime. We evaluated the model’s performance using the average precision (AP) [76] and average recall (AR) [77] metrics as implemented in the torchmetrics library (https://github.com/Lightning-AI/metrics/blob/v0.8.0/torchmetrics/detection/mean_ap.py#L133-L790) and defined as follows:

$$\begin{array}{c} \mathrm{AP}=\frac{1}{101}\sum_{r\in \left\{0,0.01,\ldots ,1\right\}}p_{\mathrm{int}\mathrm{erp}}\left(r\right),\;\text{where}\\ \;p_{\mathrm{int}\mathrm{erp}}\left(r\right)=\underset{\bar{r}:\bar{r}\geq r}{\text{max}}\;p\left(\bar{r}\right) \end{array}$$ (6)

where $p(\bar{r})$ is the precision at recall $\bar{r}$.

$$\mathrm{AR}=\frac{2}{n}\sum_{i=1}^{n}\max\left(\mathrm{IoU}\left({\mathrm{gt}}_{i}\right)-0.5,0\right)$$ (7)

where *IoU*(*gt_i_*) is the IoU between the annotation *gt_i_* and the closest detection proposal, and *n* is the number of detections per image. We reported the same AP and AR metrics as Madsen et al. [14].

We optimized the model using SGD for 32 epochs (∼24k iterations) with a batch size of 8, a weight decay of 10^−4^, and a momentum of 0.9. We selected the base learning rate by sweeping across 3 logarithmically spaced values between 3 × 10^−2^ and 8 × 10^−4^ (we searched over 3 values instead of 5 because of time constraints and limited computational resources). We increased the learning rate linearly for 1,500 steps from an initial learning rate that is 10^−3^ times the base learning rate. The learning rate was multiplied by 0.1 at the 17,000th and 22,000th steps. We trained the model using mixed precision and reported the best-performing cross-validation average.

**Wheat spikelet counting.** The wheat spikelet counting task involves estimating the number of wheat spikelets in a given image, which, if accurate, can provide better estimates of grain yield.

In this work, we used the density estimation approach as defined by Alkhudaydi and De La lglesia [78]. We used the Global Wheat Spikelet (GWS) dataset [79], which contains 200 images taken from the USASK_1 sub-dataset of the 2020 GWHD dataset [68]. Sixty-seven images were annotated for evaluating an unsupervised domain adaptation method. We split the dataset using a 5-fold cross-validation scheme.

We used the detection and regression network (DRN) proposed by Farjon et al. [16] to detect and count the wheat spikelets. The DRN model consists of a ResNet-50 [66] backbone with an FPN [72]. A fully convolutional network [80] is attached to the top-down pathway’s second to last pyramid scale. It gradually fine-tunes density map predictions in 4 independent stages to reach its final output, which is a 2D density map approximating the ground truth density map. The final density map prediction is used to obtain an initial count estimate by applying a non-maximum suppression procedure. The final count is estimated from a combination of the initial count estimate and the pooled feature vectors from the last 4-stage density map predictions. The network minimizes the weighted smooth L1 loss of the final count estimate and each of the 4 density map predictions in the detection sub-model. In our experiments, we adopted the default hyperparameters of the model [16].

We trained the model using the AdamW optimizer [81] for ∼1,400 steps (200 epochs) with a batch size of 8, a weight decay of 10^−4^, *β*_1_ = 0.9, and *β*_2_ = 0.99. We also used the PyTorch [73] implementation of ResNet-50 with an FPN backbone. We selected the base learning rate through a hyperparameter tuning procedure that swept across 5 logarithmically spaced values between 10^−3^ and 5 × 10^−5^. We used a cosine learning rate schedule with a minimum learning rate of 10^−7^. We applied only the random horizontal flip augmentation during training and no augmentation during testing. Due to the relatively small size of the datasets for the counting tasks, we did not drop the last incomplete batch when the dataset size was not evenly divisible by the batch size. Following previous work [79], we evaluated performance using the root mean square error (RMSE), mean absolute error (MAE), and the coefficient of determination (*R*^2^). We trained the model using mixed precision and reported the best-performing cross-validation average.

**Leaf counting.** The leaf counting task has been one of the most influential tasks in image-based plant phenotyping owing to the long-running leaf counting challenge (LCC) introduced by the Computer Vision Problems in Plant Phenotyping (CVPPP) workshop in 2016. The task involves estimating the number of leaves in a given plant image. The number of leaves on a plant can provide clues about the health of the plant and its growth rate.

This study used the Juelich Challenges version of the CVPPP2017 LCC dataset [82–84]. The original dataset from CVPPP2017 includes 4 sub-datasets labeled A1 to A4. The A3 sub-dataset contains 27 training images of tobacco plants, while the A1, A2, and A4 sub-datasets contain 128, 31, and 624 training images of Arabidopsis plants, respectively. The Arabidopsis images have an average resolution of 450 × 460, while the A3 sub-dataset has a resolution of 2,448 × 2,048. All images in the dataset are captured under controlled lighting conditions and contain a single plant. A different version of the dataset was introduced in the 2021 Computer Vision in Plant Phenotyping and Agriculture workshop for the LCC hosted on the Helmholtz Data Challenges (https://helmholtz-data-challenges.de/web/challenges/challenge-page/85/overview) platform. In this version, all the training images from the 4 sub-datasets were combined into one and then split into 90% training and 10% validation sets. The annotations in the original dataset are in the forms of the raw leaf counts and binary images where the leaf centroid is marked as a single pixel. Only the leaf counts are provided in the Juelich Challenges version.

We used a similar implementation for this task as the wheat spikelet counting task. The main differences are that we used a batch size of 32 and did not use the cross-validation scheme for this task since it has a fixed validation set. We selected the base learning rate through a hyperparameter tuning procedure that swept across 5 logarithmically spaced values between 10^−4^ and 10^−6^. We also resized the images to a resolution of 512 × 512 during training and testing. Additionally, during training, we applied random horizontal flip augmentation. Since we used the density estimation approach and it requires dot annotations, we added the leaf centroid annotations from the CVPPP2017 dataset to the Juelich Challenges version. We used the LCC metrics to evaluate performance. These metrics include count difference, MAE, mean square error, and count agreement. We trained the model using mixed precision and reported the results of the best-performing model on the validation and test sets. We reported only the MAE metric for the test results since that is the only metric available in the ongoing LCC.

### Implementation details

We used a ResNet-50 model as the encoder for all the representation learning tasks in this study, including pretraining and downstream tasks. The training was done on 4 Nvidia Tesla V100 Graphics Processing Units (GPUs) with a batch size of 256 for all pretraining methods (the downstream tasks were trained on a single GPU). To minimize the influence of dataset size at the pretraining stage, we adjusted the training steps for each dataset to match a standard so that the model “sees” the same number of images during training. We used ∼1 million steps as the standard, which is approximately the number of iterations when training on ImageNet for 200 epochs at a batch size of 256. We maintained the same batch size for all pretraining datasets. All tasks were implemented with the PyTorch framework [73] and trained with mixed precision. We used Weights & Biases [85] to track experiments and perform hyperparameter sweeps.

Due to the combinatorial nature of our experiment design and limited computational resources, we did not do any hyperparameter tuning at the pretraining stage. We relied instead on the default hyperparameters for each method, which are tuned on the ImageNet [20] dataset. We, however, made some minor modifications. For supervised pretraining, since we adjusted the batch size from 2,048 to 256, we scaled the learning rate by the square root of 256/2,048. For both MoCo v2 and DenseCL pretraining, we replaced all the BN layers with synchronized batch normalization (syncBN) layers. SyncBN normalizes across all GPUs instead of per GPU, and its performance is on par with per-device BN [86].

### Representation similarity analysis

Representation similarity analysis enables the quantitative comparison of internal representations between neural networks. Several similarity indices exist for this purpose, including canonical correlation analysis [87], centered kernel alignment (CKA) [88], and orthogonal Procrustes distance [89].

Although linear CKA is the common approach for measuring similarity between neural networks, here we used the orthogonal Procrustes distance. The main reason for this is that the orthogonal Procrustes distance is more robust than linear CKA, especially with respect to sensitivity to slight differences between representations [90]. The orthogonal Procrustes distance measures the difference between 2 sets of vectors after an orthogonal transformation (from the solution of the orthogonal Procrustes problem [89]) has been applied to map one set of vectors as closely as possible to the other. Like CKA, it is invariant to isotropic scaling and orthogonal transformations [91]. These are desirable properties for a similarity index [88]. The orthogonal Procrustes distance has been shown to overestimate similarity when the number of data examples is low [91], so we make sure to use a large number of samples in computing the similarity.

To compute the similarity metric, we extracted the output of each residual block of the encoder after a forward pass. We reduced the extracted 3-dimensional feature maps to a 1-dimensional feature vector for computational efficiency by applying a global average pooling operation. To compute the orthogonal Procrustes distance, we used 25,000 examples drawn from the test set of each of the pretraining datasets. The result of the orthogonal Procrustes distance is usually a value between 0 and 2. However, we followed the method of Bonheme and Grzes [91] to produce a similarity score in the range of 0 (dissimilar) and 1 (similar).

## Results

Through the large set of experiments described in Materials and Methods, we found that data redundancy, pretraining method, and pretraining domain had varying degrees of impact on the performance of the different downstream plant phenotyping tasks.

### Effects of data redundancy

**Fig. 2.**
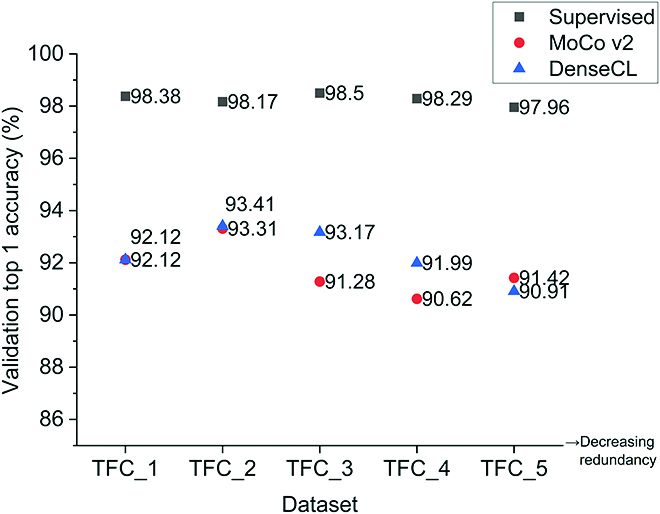
Top 1 classification accuracy on the validation set of the TerraByte Field Crop (TFC) dataset when trained with different methods and sub-datasets with varying amounts of spatial redundancy. We used the linear evaluation protocol to obtain the results for the MoCo v2 and DenseCL.

We studied the effects of data redundancy on the quality of the learned representations and the downstream performance by creating 5 subsets of the TFC dataset with varying degrees of redundancy. Redundancy, in this case, means that some images or parts of images occur multiple times within the sub-dataset due to the substantial spatial overlap from the data generation process. We applied each of the sub-datasets to each of the pretraining methods. For the self-supervised pretraining methods, we used the linear evaluation protocol to evaluate the learned representations on the validation set of the dataset. In that case, we fine-tuned each of the sub-datasets for ∼4,900 steps. For each pretraining method, we report the top 1 accuracy on the validation set of the TFC dataset in Fig. 2. We further fine-tuned the pretrained models on each downstream task and reported the results in Table S1.

From Fig. 2, we observe that supervised representations have superior linear classification performance to the SSL representations, regardless of the level of data redundancy. This result is contrary to recent works (like [62]) that show SSL methods outperforming supervised pretraining on classification tasks. We further observe that, while performance tends to decrease as redundancy decreases, the performance of the models trained in a supervised manner is remarkably stable across the 5 degrees of redundancy, with less than a 1% difference between the best and worst accuracy scores, compared to the self-supervised models, whose performance can vary by as much as 3%. One reason for the stability in the performance of the supervised models could be that the training recipe for the supervised pretraining method includes stronger data augmentation than the self-supervised methods. The stronger data augmentation pipeline potentially generates more novel samples during training, reducing the number of redundant examples that the model learns from.

Figure 2 also shows that the linear classification performance of MoCo v2 and DenseCL follows a similar trend, with a slight increase in performance when redundancy is decreased between the TFC_1 and TFC_2 sub-datasets, and a drop in performance as redundancy is further decreased beyond that. This trend suggests that some level of redundancy may be desirable in terms of the absolute accuracy score—there is a 75% overlap between consecutive frames in the TFC_2 sub-dataset, but training on it with both MoCo v2 and DenseCL achieves the highest linear classification accuracy, relative to the other sub-datasets. On the other hand, the relative difference in performance between the best-performing dataset (TFC_2) and the dataset with the least redundancy (TFC_5) is about 3% for DenseCL and 2% for MoCo v2. Although the design of our study does not allow us to determine if these differences are statistically significant, in practical terms, the difference may not be large enough to justify additional compute costs associated with training on a more redundant dataset.

To test the influence of redundancy in the pretraining dataset on the performance of downstream tasks, we fine-tuned models trained with the 5 sub-datasets on the 4 downstream tasks. The results, which are reported in Table S1, show that redundancy in the pretraining dataset has a small effect on the performance of the downstream tasks. There are some cases, like the plant instance detection task, where the difference in performance between the best and worst sub-dataset is in the last significant digit. In other cases, the difference in performance between the best and worst sub-dataset is more apparent. Examples of such cases include using MoCo v2 with the TFC_5 dataset on the wheat head detection task (as measured by the ADA metric) or using the supervised pretraining method with TFC_5 on the leaf counting task (as measured on the validation and test sets).

Overall, the results of these experiments suggest that the SSL methods are more sensitive to redundancy in the pretraining dataset, particularly on the linear classification task. We selected the TFC_5 sub-dataset for further experiments since it contained the least amount of redundancy and did not result in severe performance degradation across different tasks.

### Transfer learning

In the following sections, we show the results of our experiments on the transfer of representations learned from MoCo v2, DenseCL, and supervised pretraining to 4 downstream tasks. Our experiments explored the influence of the pretraining method and the image domain of the pretraining dataset on the transfer performance.

#### Wheat head detection

**Fig. 3.**
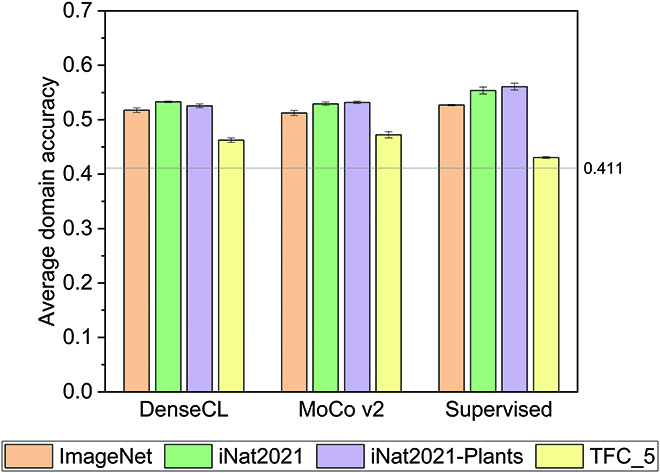
The mean average domain accuracy (ADA) for the wheat head detection task on the GWHD_2021 dataset after fine-tuning the last 3 layers of a Faster-RCNN model initialized with weights from different pretrained models. The horizontal line indicates the value of the metric when the model is trained from random initialization (0.411). See Table S2 for a more detailed report of the transfer performance, including other metrics.

Figure 3 shows that pretraining with the TFC_5 dataset results in the worst performance for each pretraining method. This is surprising since the image domain of the TFC_5 dataset is closest to the GWHD_2021 dataset as shown in Fig. S4. Possible explanations for this include the low diversity of the images in the TFC_5 dataset and the fact that the samples in the dataset contain multiple objects of interest with similar appearances, unlike popular object-centric datasets like ImageNet [20], which usually contains a distinct object near the center of the image. The other 3 pretraining datasets follow an expected trend regarding the relationship between the image domain of the source task and the downstream performance. Pretraining with the iNat2021-Plants dataset leads to the best performance, followed by the iNat2021 and ImageNet datasets, in the order of increasing domain distance. Although this trend breaks with DenseCL for the ADA metric, it is consistent for the AP metrics, as shown in Table S2. The ADA metric suggests that pretraining with DenseCL on the iNat2021-Plants dataset is more susceptible to domain shift than on the iNat2021 dataset. Figure S5 shows that the largest difference in accuracy between the 2 methods occurs with the TERRAREF_1 and TERRAREF_2 sub-datasets.

Figure 3 also shows that supervised pretraining outperforms self-supervised pretraining in most cases. Although the trend holds with the AP metrics as shown in Table S2, the TFC_5 dataset presents an exception, where transfer performance, as measured by the ADA metric, is higher with self-supervised pretraining than with supervised pretraining. The exception to the trend can also be observed in the experiments for data redundancy as shown in Table S1, suggesting that the self-supervised representations from the TFC_5 dataset are more robust to domain shift when fine-tuned on the wheat head detection task. This is supported by Fig. S6, which shows substantial gaps in the domain accuracy between supervised and SSL pretrained models. It is worth noting that even though the supervised pretraining method outperforms the self-supervised pretraining methods in most cases, the self-supervised pretraining methods outperform training from random initialization in all cases.

Table S2 shows that DenseCL has a slight edge over MoCo v2, particularly on the AP metrics. However, this difference may not be practically meaningful. The difference in performance between the 2 methods is smaller than the difference between the supervised pretraining method and the SSL methods.

Table S2 also shows that the modifications we made to the original implementation of MoCo v2 [24] and DenseCL [32] (replacing BN layers with syncBN layers) resulted in worse performance for both methods.

#### Plant detection

As shown in Table 2, training from scratch for this task is a strong baseline, outperforming most of the pretrained models, including the baseline model from Madsen et al. [14], which was a Faster-RCNN model without an FPN pretrained on the COCO dataset [21]. This is also true for the AR metrics, reported in Table S3. However, for all the pretraining methods, pretraining with the iNat2021-Plants dataset matches or exceeds the random initialization baseline. So does pretraining with the standard supervised learning method across the 4 pretraining datasets. The overall best performance for this task is achieved by fine-tuning a model that is pretrained in a supervised manner with the iNat2021-Plants dataset.

**Table 2.** Detection performance on the OPPD dataset for different initialization schemes. We report the mean (SD) for each metric. The overall best score for each metric is **bolded**, while the best score for each pretraining method is underlined.

Method	No. of epochs	*AP*	_*AP*50_	_*AP*75_
Random init	–	0.423 (0.040)	0.683 (0.062)	0.457 (0.046)
**Supervised**				
COCO [14]	–	0.370 (0.024)	0.655 (0.045)	0.373 (0.026)
ImageNet-1k [PyTorch]	90	0.424 (0.039)	0.677 (0.062)	0.459 (0.044)
ImageNet-1k [ours]	200	0.429 (0.042)	0.689 (0.066)	0.465 (0.045)
iNat2021	95	0.431 (0.041)	0.689 (0.062)	0.469 (0.045)
iNat2021-Plants	223	**0.438 (0.041)**	**0.695 (0.062)**	**0.471 (0.046)**
TFC_5	1,668	0.425 (0.039)	0.687 (0.063)	0.459 (0.042)
**MoCo v2**				
ImageNet-1k [25]	200	0.401 (0.035)	0.661 (0.058)	0.432 (0.039)
ImageNet-1k [ours]	200	0.414 (0.038)	0.671 (0.059)	0.445 (0.041)
iNat2021	95	0.417 (0.037)	0.672 (0.059)	0.449 (0.042)
iNat2021-Plants	223	0.423 (0.039)	0.681 (0.059)	0.455 (0.043)
TFC_5	1,668	0.412 (0.038)	0.670 (0.058)	0.428 (0.041)
**DenseCL**				
ImageNet-1k [32]	200	0.424 (0.040)	0.678 (0.064)	0.458 (0.045)
ImageNet-1k [ours]	200	0.412 (0.038)	0.670 (0.058)	0.442 (0.043)
iNat2021	95	0.420 (0.038)	0.678 (0.060)	0.450 (0.044)
iNat2021-Plants	223	0.424 (0.040)	0.682 (0.059)	0.456 (0.045)
TFC_5	1,668	0.412 (0.038)	0.670 (0.058)	0.441 (0.043)

Table 2 also shows that, regardless of the pretraining method, fine-tuning a model that is pretrained with the TFC_5 dataset results in the worst performance for this task, even though the image domain of the TFC_5 dataset is closest to the OPPD dataset as shown in Fig. S4. Furthermore, the results show that there is little difference in performance between the MoCo v2 and DenseCL methods across all pretraining datasets. This finding is consistent with the results in Table S1 for the plant instance detection task. In Table 2, we observe the largest difference in performance between the 2 methods in the *AP*_75_ metric reported for the TFC_5 dataset, which is 0.013 points lower for the MoCo v2 compared DenseCL.

Finally, Table 2 shows that the modifications we made to the original implementation of MoCo v2 [24] improved the downstream performance while our changes to the DenseCL [32] model did not.

#### Wheat spikelet counting

Table 3 shows that the supervised pretraining method outperforms the SSL methods when transferring learned representations to the wheat spikelet counting task. This finding is supported by the results reported in Table S1 for the wheat spikelet counting task. Neither result reveals any noticeable advantages to using DenseCL over MoCo v2 for this task, at least in terms of the MAE between the actual and predicted wheat spikelet counts. Table 3 also highlights the superior performance of SSL pretraining over the unsupervised domain adaptation method proposed by Ayalew et al. [79]. Both methods are able to leverage unlabeled datasets; however, the SSL methods offer a more general framework for utilizing a diverse pool of unlabeled data.

**Table 3.** Transfer results for the wheat spikelet counting task. We show the overall best score for each metric in **bold letters** and underline the best score for each pretraining method. For each metric, ↓ means lower values are better, while ↑ means higher values are better.

**Method**	**No. of epochs**	**MAE** ↓	**RMSE** ↓	***R***^**2**^ ***↑***
Random init	–	28.6 (4.2)	37.5 (4.7)	0.58 (0.12)
Unsupervised domain adaptation [79]	–	29.5	35.8	0.66
**Supervised**				
ImageNet-1k [PyTorch]	90	22.1 (5.0)	28.6 (6.1)	0.69 (0.22)
ImageNet-1k [ours]	200	21.0 (5.2)	29.0 (6.2)	0.75 (0.07)
iNat2021	95	22.1 (3.9)	28.8 (2.8)	0.74 (0.11)
iNat2021-Plants	223	21.9 (5.4)	28.5 (5.1)	0.76 (0.07)
TFC_5	1,668	**20.1 (2.5)**	**27.1 (4.9)**	**0.78 (0.06)**
**MoCo v2**				
ImageNet-1k [24]	200	22.7 (3.9)	29.2 (3.9)	0.74 (0.09)
ImageNet-1k [ours]	200	25.7 (4.9)	31.5 (7.3)	0.67 (0.21)
iNat2021	95	25.1 (6.1)	30.9 (4.7)	0.70 (0.14)
iNat2021-Plants	223	24.2 (3.0)	32.0 (3.1)	0.63 (0.29)
TFC_5	1,668	24.6 (6.0)	31.9 (9.3)	0.66 (0.25)
**DenseCL**				
ImageNet-1k [32]	200	22.1 (5.0)	28.6 (6.1)	0.69 (0.22)
ImageNet-1k [ours]	200	25.6 (8.4)	31.0 (8.1)	0.70 (0.14)
iNat2021	95	26.5 (6.1)	33.9 (7.8)	0.57 (0.33)
iNat2021-Plants	223	25.1 (8.8)	32.8 (7.2)	0.67 (0.13)
TFC_5	1,668	21.7 (5.9)	27.8 (6.6)	0.75 (0.15)

MAE, mean absolute error; RMSE, root mean square error.

Unlike the detection tasks, pretraining with the TFC_5 dataset has a strong positive influence on this task, as shown in Table 3—it achieves the best performance for both supervised and DenseCL methods. On the other hand, the results show that the iNat2021 dataset is among the worst-performing pretraining datasets for this task. A possible explanation for the observed performance differences is that the models for the detection and counting tasks use the FPN [72] in different ways. Faster R-CNN [71] makes a prediction using all the feature maps in the top-down pathway, whereas the DRN [16] model uses only the second-to-last feature map from the top-down pathway of the FPN, which combines both high-resolution and semantically meaningful features. In other words, the DRN model is more sensitive to the quality of the features in that particular feature map.

Similar to the results of the wheat head detection task, Table 3 shows that using the original implementations of MoCo v2 [24] and DenseCL [32] results in better performance than our modified versions.

#### Leaf counting

Based on the values for the MAE reported in Table 4 for the validation set, supervised pretraining generally outperforms self-supervised pretraining for the leaf counting task. However, this result does not carry over to the test set, where DenseCL has a slight edge over supervised pretraining, unlike the results of the other downstream tasks in this study. Our results on the test set are outperformed by that of Farjon et al. [16], who used the same model as we did and trained from scratch with a different training procedure.

**Table 4.** The transfer performance on the leaf counting task. We show the overall best score for each metric in bold letters and underline the best score for each pretraining method. For each metric, ↓ means lower values are better, while ↑ means higher values are better.

Method	No. of epochs	Validation				Test
		CountDiff ↓	MAE ↓	MSE ↓	Count Agreement (%) ↑	MAE ↓
Random init	–	0.14	0.73	1.40	45.9	1.35
Farjon et al. [16] (DRN)	–	–	–	–	–	0.72 (0.81)
**Supervised**						
ImageNet-1k [PyTorch]	90	-0.08	0.70	1.16	47.3	0.91
ImageNet-1k [ours]	200	0.14	0.73	1.54	50.0	0.91
iNat2021	95	0.04	**0.61**	**1.01**	54.0	0.89
iNat2021-Plants	223	0.11	0.68	1.19	48.6	1.05
TFC_5	1,668	0.18	0.66	1.23	48.6	0.97
**MoCo v2**						
ImageNet-1k [24]	200	0.18	0.69	1.12	45.9	0.85
ImageNet-1k [ours]	200	0.12	0.72	1.53	51.4	0.99
iNat2021	95	**0.03**	0.65	1.11	52.7	0.95
iNat2021-Plants	223	0.14	0.76	1.49	47.3	0.92
TFC_5	1,668	0.12	0.80	1.85	45.9	1.10
**DenseCL**						
ImageNet-1k [32]	200	-0.08	0.70	1.16	47.3	0.91
ImageNet-1k [ours]	200	0.08	0.70	1.46	**54.1**	0.88
iNat2021	95	0.09	0.69	1.23	51.4	0.93
iNat2021-Plants	223	0.04	0.72	1.26	50.0	**0.83**
TFC_5	1,668	0.15	0.69	1.58	47.3	1.09

CountDiff, count difference; MSE, mean square error; CountAgreement, count agreement.

Table 4 also shows that pretraining with the iNat2021-Plants dataset yields the best result on the test set for the leaf counting task, while the TFC_5 dataset is among the worst-performing pretraining dataset. Interestingly, the results for both the leaf counting and the wheat spikelet counting tasks show that the ImageNet dataset is among the top-performing pretraining datasets (it is usually second-best). These results are surprising because the ImageNet dataset is the farthest from the LCC and GWS datasets in terms of domain distance, and we expected it to be the case that the farther the domain distance, the worse the performance. Considering that the DRN model uses the second-to-last feature map from the top-down pathway of the FPN [72], which contain both high-resolution low-level features and semantically meaningful high-level features, the results suggest that the high-resolution low-level features play a bigger role in the performance of the counting tasks compared to the high-level semantic features.

Finally, Table 4 shows that the modifications we made to the original implementation of DenseCL [32] improved the downstream performance while our changes to the original implementation of MoCo v2 [24] did not.

### Representation similarity analysis

**Fig. 4.**
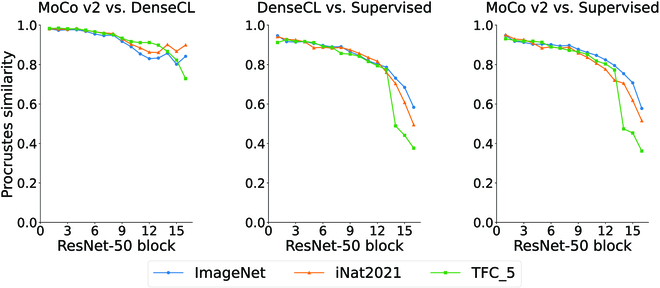
The figure shows the Procrustes similarity between the output of each block of a ResNet-50 encoder for different combinations of pretraining datasets and pretraining algorithms. The *x*-axis shows the Procrustes similarity ranging from 0 (dissimilar) to 1 (similar) for each corresponding ResNet-50 block. We show the full matrix of similarity scores in Fig. S7, i.e., the similarity between the output of each block in one network and the output of every block in the other network. This figure shows the diagonal of those matrices.

To understand the differences in the representations produced by the different pretraining methods, we compute the Procrustes similarity between the output of each ResNet block for each pair of pretraining methods. We did not use the iNat2021-Plants dataset for this analysis because we could not sample the test set for images belonging only to the Plants supercategory. The results are shown in Fig. 4. We include the results of using linear CKA in Figs. S8 and S9, which show a similar trend to the Procrustes similarity.

Figure 4 shows that MoCo v2 and DenseCL learn similar representations (Procrustes similarity score above 0.8) throughout the network for the 3 datasets. On the other hand, the dissimilarity between supervised and SSL representations grows toward the final layers of the network. This difference is likely related to the loss function being optimized by the 2 methods—whereas the cross entropy loss maps an input to a known class, the contrastive loss is used to learn a similarity metric for comparing inputs in an embedding space in an augmentation-invariant manner.

## Discussion

The key question we investigated in this study is as follows: How effective is self-supervised pretraining compared to supervised pretraining for image-based plant phenotyping tasks? We studied this question in the context of 4 plant phenotyping tasks: wheat head detection, plant instance detection, wheat spikelet counting, and leaf counting. Our analysis focused on the influence of 2 main factors on the performance of the downstream tasks: the pretraining method and the domain of the pretraining dataset. Secondarily, we studied the effects of redundant images in the TFC dataset on the quality of representations learned by the pretrained models and the performance of the downstream tasks.

We found that supervised pretraining is more effective than self-supervised pretraining for the downstream tasks we studied, except for the leaf counting task. This means that the best-performing model for each task, besides the leaf counting task, is a model pretrained with the supervised method. In the case of the leaf counting task, the best-performing model was pretrained with the DenseCL method.

Our results add to the list of studies that show that supervised pretraining is more effective than self-supervised pretraining. This includes the works of Van Horn et al. [36] and Cole et al. [54], which show that standard supervised pretraining outperforms SimCLR [23] (an SSL method) for fine-grained image classification tasks. Our results also contrast with the findings of some other benchmarking studies. For example, Mensink et al. [18] report that fine-tuning a model trained with SimCLR on ImageNet outperforms fine-tuning a model trained with the supervised method on ImageNet. Similarly, Kotar et al. [51] find that, for 18 out of 20 diverse downstream tasks, the best-performing self-supervised pretrained models outperform a model trained with ImageNet in a supervised manner, and Ericsson et al. [52] report that the best self-supervised methods can outperform supervised pretraining on most of the downstream tasks they studied. However, the analyses of these studies are usually centered around the ImageNet dataset in some way. Both Mensink et al. [18] and Ericsson et al. [52] compare the performance of models pretrained with the supervised method on ImageNet with models pretrained with self-supervised methods on ImageNet. Kotar et al. [51], on the other hand, compare the performance of a model pretrained with the supervised method on ImageNet with the best-performing models pretrained with 1 of 3 self-supervised methods on 4 different datasets. In contrast, our analysis considers multiple pretraining methods and pretraining datasets for each downstream task.

One reason we suspect that supervised pretraining is more effective in our study is that the training recipe we used for the supervised method includes a stronger data augmentation pipeline and has been more extensively tuned (on the ImageNet dataset) than the training recipe that is most commonly used for the supervised method in the literature. Evidence of this can be seen in Tables 3 and 4 and Tables S2 and S3, which show that our supervised ImageNet-pretrained models match or outperform the default ImageNet-pretrained model provided by the PyTorch library.

The representation similarity analysis we performed using the Procrustes similarity score may also provide further insight into the differences between the performance of the supervised and self-supervised models. The results show a high degree of similarity (Procrustes score greater than 0.8) between representations learned by DenseCL models and those learned by MoCo v2, which may explain why we found only a slight difference in performance between the 2 methods in the downstream tasks. On the other hand, comparing the representation similarity of supervised and self-supervised models shows a trend in which, relative to the early blocks, there is a large and sharp drop in similarity in the last few blocks. The work of Grigg et al. [92] shows a similar trend when comparing the similarity of SimCLR representations to supervised representations (both trained on the CIFAR-10 [93] dataset) using the linear CKA method. In related work, Neyshabur et al. [94] show that the features learned in the early layers are more general and more likely to be reused during transfer learning, while the features learned in the higher layers are more specialized to the task at hand and more sensitive to changes in their parameters. In addition to the high similarity between supervised and self-supervised representations in the early layers, feature reuse helps explain why both methods are successful in the first place—they can both learn general features in the early layers, which are reused in downstream tasks. However, it does not explain why the supervised method outperforms the self-supervised methods in our study. We conjecture that the models trained with the supervised method learn stronger semantic representations in the deeper layers than those trained with the self-supervised method, hence the divergence in representation similarity. We note that all the models we used in the downstream tasks have an FPN [72] attached, which aims to semantically enrich the representations in the shallower layers using information from the deeper layers. The FPN is the likely mechanism through which the downstream tasks take advantage of the semantically rich representations in the deeper layers.

On the influence of the pretraining dataset, we corroborate previous studies [18,51,54] in showing that using a pretraining dataset in the same or similar domain as the downstream dataset typically results in the best performance on the downstream task. We observed that pretraining with the iNat2021-Plants dataset resulted in a better downstream performance than pretraining with the TFC_5 dataset, especially on the wheat head and plant instance detection tasks. This is particularly surprising because the TFC_5 dataset has the closest domain distance to all the downstream datasets used in this study. We suspect that the diversity of the iNat2021-Plants dataset is one of the factors that give it an edge over the TFC_5 dataset—the iNat2021-Plants dataset has 4,271 different classes of plants, in stark contrast to the 5 crop categories of the TFC_5 dataset, which also have high intra-class similarity. It could also be that our attempt to control for the difference in the dataset size did not work as expected—the iNat2021-Plants dataset has 1.1 million samples, compared to the 153,627 samples in the TFC_5 dataset and there is a chance of overfitting on the smaller dataset with a longer training schedule. However, it is worth noting that pretraining with the TFC_5 dataset is particularly beneficial for the wheat spikelet counting task.

Our results show that the supervised pretraining method is less sensitive to redundancy in the TFC dataset than the SSL methods, particularly in the context of linear classification. We also found that the redundancy in the TFC dataset has a small effect on performance in the 4 downstream tasks. These results highlight the importance of paying attention to dataset redundancy when training models for plant phenotyping tasks, especially when using SSL methods.

We note that, in most cases, fine-tuning self-supervised representations on the downstream tasks performs better than training from random initialization, which can benefit applications where domain-specific data are required. The advantage of SSL, in that case, is a reduction in the number of annotations needed.

Although our study shows the promise of SSL for image-based plant phenotyping tasks, it has several potential limitations. Firstly, our analysis and conclusions are based on empirical observations with few theoretical justifications. Secondly, the lack of hyperparameter tuning at the pretraining step means that results using datasets other than ImageNet may not be optimal. In most of the cases where we have modified the original implementation of MoCo v2 [24] and DenseCL [32], the results are not as good as those reported in the original papers, and the difference in performance is sometimes more drastic than expected based on the results of the study that inspired the changes [86]. Part of the reason for this is the difficulty of reproducing results of deep learning studies, which are often highly sensitive to hyperparameters and implementation details. This points to the need for more rigorous hyperparameter tuning, particularly when applying published methods to new datasets. Nonetheless, we note that pretraining with ImageNet did not always result in the best downstream performance in this study despite being more rigorously tuned for each pretraining method. In other words, there may be more room for improvement in the pretraining step for the other datasets used in this study, which we plan to explore in future work. Finally, we only explored a few conditions/variables that may affect transfer performance, limiting the generalization capacity of our conclusions. Our experiments on dataset redundancy was done at the level of full video frames and ignored the redundancy that may exist after splitting the frames into patches. We also did not explore the effect patch overlap, which may also contribute to redundancy. Therefore, we recommend that future work conduct more in-depth analysis of the effect of dataset redundancy in the context of plant phenotyping.

Future work could extend our study to explore datasets from crop breeding trials, which are even more fine-grained than the crop dataset explored in this work. Our analysis could also be extended to explore the differences in contrastive and non-contrastive learning methods (e.g., BYOL [27]). While we showed that self-supervised and supervised models learn relatively different representations in the last few layers, future work could explore what is being learned to better understand why supervised learning is currently superior and how SSL methods can be improved. Some promising attempts to improve SSL can be explored for plant phenotyping, such as improving hard positive mining for the contrastive loss [95], incorporating metadata into SSL [96–98], and combining multiple data modalities [99,100]. SSL can potentially be used to learn a richer representation of plant phenotypes by aligning it with genotype and environment data in a joint embedding space.

## Data Availability

All the datasets used in this study are either publicly available or available upon request from their respective sources.

## Supplementary Materials

Supplementary 1 Materials and Methods Fig. S1. Conceptual illustration of the MoCo v2 pipeline. Fig. S2. The pipeline for the dense contrastive learning framework. Fig. S3. A schematic of the canonical supervised learning pipeline. Fig. S4. The optimal transport cost between the representations of the pretraining datasets and the downstream datasets. Fig. S5. The accuracy for each domain/sub-dataset of the GWHD_2021 dataset when fine-tuning models pretrained with DenseCL on the iNat2021 and iNat2021-Plants datasets. Fig. S6. The accuracy for each domain/sub-dataset of the GWHD_2021 dataset when fine-tuning models pretrained on the TFC_5 dataset with different methods. Fig. S7. The Procrustes similarity metric between the output of ResNet-50 blocks from different pretrained models. Fig. S8. Linear CKA value between the output of ResNet-50 blocks from different pretrained models. Fig. S9. Linear CKA between the output of corresponding ResNet-50 blocks from different pretrained models. Table S1. The table shows the performance when an encoder pretrained on the subsets of the TFC dataset is fine-tuned on the downstream tasks. Table S2. Wheat head detection performance with the GWHD_2021 dataset for different initialization schemes. Table S3. The transfer performance on the plant instance detection task with the OPPD dataset.
