# Supplementary material for: Benchmarking Self-Supervised Contrastive Learning Methods for Image-Based Plant Phenotyping
Source: Plant Phenomics. 2023 Apr 3;5:0037. doi: 10.34133/plantphenomics.0037 (PMC10079263; doi:10.34133/plantphenomics.0037)
Supplement: Supplementary 1 — Materials and Methods Fig. S1. Conceptual illustration of the MoCo v2 pipeline. Fig. S2. The pipeline for the dense contrastive learning framework. Fig. S3. A schematic of the canonical supervised learning pipeline. Fig. S4. The optimal transport cost between the representations of the pretraining datasets and the downstream datasets. Fig. S5. The accuracy for each domain/sub-dataset of the GWHD_2021 dataset when fine-tuning models pretrained with DenseCL on the iNat2021 and iNat2021-Plants datasets. Fig. S6. The accuracy for each domain/sub-dataset of the GWHD_2021 dataset when fine-tuning models pretrained on the TFC_5 dataset with different methods. Fig. S7. The Procrustes similarity metric between the output of ResNet-50 blocks from different pretrained models. Fig. S8. Linear CKA value between the output of ResNet-50 blocks from different pretrained models. Fig. S9. Linear CKA between the output of corresponding ResNet-50 blocks from different pretrained models. Table S1. The table shows the performance when an encoder pretrained on the subsets of the TFC dataset is fine-tuned on the downstream tasks. Table S2. Wheat head detection performance with the GWHD_2021 dataset for different initialization schemes. Table S3. The transfer performance on the plant instance detection task with the OPPD dataset. [file plantphenomics.0037.f1.pdf]

Supplementary Materials

A Materials and Methods

A.1 TFC Data Curation

The TerraByte Field Crop (TFC) data is an extensive image dataset of common Canadian crops and weeds, taken in indoor and field settings for computer vision tasks in the agricultural domain [37]. The data consists of 402 videos recorded from various crop fields in the growing seasons of 2019 (June to July) and 2020 (May to August). The data from the 2020 growing season included images from soybean, faba bean, wheat, oats, and canola fields, whereas only soybean fields were recorded in the 2019 season. All videos were recorded using a ZED camera, a stereo camera with two lenses capable of depth sensing. Only frames from the left lens are used. The camera can also record at 15 FPS at a resolution of 2208×1242 for each lens or 60 FPS at a resolution of 1280×720. To construct the original dataset, the researchers extracted all the frames from the videos. At a frame rate of 60 FPS or even 15 FPS, consecutive frames are likely to be near-duplicates resulting in redundancies in the dataset. We investigated the effects of such redundancy on the transferability of representations learned using three different representation learning methods.

We started by selecting only the videos recorded in 2020 since they include metadata about the data collection process, such as date of capture, camera resolution, the height of the camera above the ground, an estimate of the camera angle and an estimate of the travel speed of the vehicle from which the video was captured. We excluded three videos from the dataset that contain corrupt frames. One of the excluded videos was taken in the Canola field (recorded on July 13, 2020), while the other two are videos of a Soybean field (recorded on May 27, 2020, and June 17, 2020). Prior to extracting frames from the videos, we calculated the ground sampling distance (GSD) as follows:

$$GSD_{height} = \frac{h_c * h_s}{f * h_f} \tag{8}$$

where h_c is the height of the camera from the ground in centimetres (cm), h_s is the height of the camera sensor in cm, f is the focal length of the camera in cm, and h_f is the height of the frame in pixels. Knowing the GSD allows us to estimate the image footprint on the ground by mapping pixels to ground distance.

For each video, we extracted frames based on the desired percentage of overlap between successive frames. The frame interval is determined using the following formula:

$$F_{int} = \max\left(1, \left\lceil \frac{GSD_{height} * h_f * 0.01 * (1-p)}{s} * fps \right\rceil\right) \tag{9}$$

where F_{int} is the frame interval, p is the desired percentage of spatial overlap between successive frames, GSD_{height} is the ground sampling distance in $cm/pixel$, h_f is the height of the frame in pixels, s is the travel speed of the vehicle in $meters/second$ and fps is the frame rate of the camera in frames per second. Extracting every F_{int}-th frame from the video roughly corresponds to shifting the camera such that the overlap between each successive photo is roughly p% along the height

of the image. We applied a 2$\times$ upsampling with bicubic interpolation to every frame that had a resolution of 1280×720.

We discarded frames that had little to no vegetation in them. To do this, we used the following vegetation index:

$$ExGr - ExR > 0 \tag{10}$$

where

$$ExGr = 2G - R - B$$

and

$$ExR = 1.4R - B$$

. The vegetation threshold was taken as the 99th percentile value of the vegetation index for all frames in videos recorded on May 27, 2020. This being early in the planting season, the plants were still in the early stages of growth, and some had not even sprouted.

We extracted all the frames from videos to construct the `TFC_1` dataset by setting $p = 1$ in eq 9. That resulted in a total of 376851 images, which we split into training (302585 images), validation (33417 images) and testing (40849 images) sets. For reference, the original dataset contained a total of 542177 images. We split each image in the training set into eight 640×640 non-overlapping patches in a 2×4 grid. The validation and test sets were not split into patches and were kept at their original resolution. Before splitting, each image was upsampled to a resolution of 2560×1440 using bilinear interpolation (only if the image was not already at that resolution). We discarded patches with little to no vegetation using the vegetation index described above. For this step, we selected a vegetation threshold of 0.1. After splitting and discarding patches, we end up with 1696889 images in the training set. The `TFC_2`, `TFC_3`, `TFC_4` and `TFC_5` sub-datasets were derived by extracting frames from the video with $p = 0.75$, $p = 0.5$, $p = 0.25$ and $p = 0$ in eq 9, respectively.

A.2 Schematic of Pre-training Methods

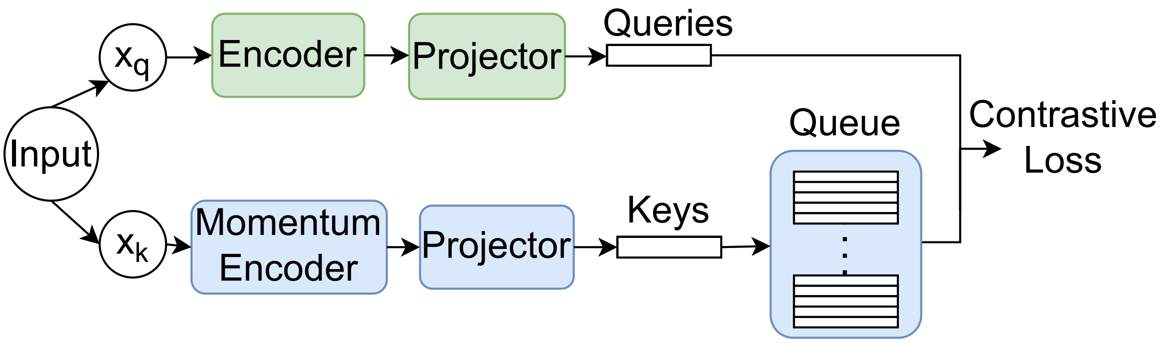

Figure S1: Conceptual illustration of the MoCo v2 pipeline. The encoder is a ResNet-50 network that ends with a global average pooling layer. The projector is a 2-layer fully-connected network with a ReLU activation function between the layers. The queries and keys are 128-dimensional vectors. The queue stores key vectors of previously seen examples.

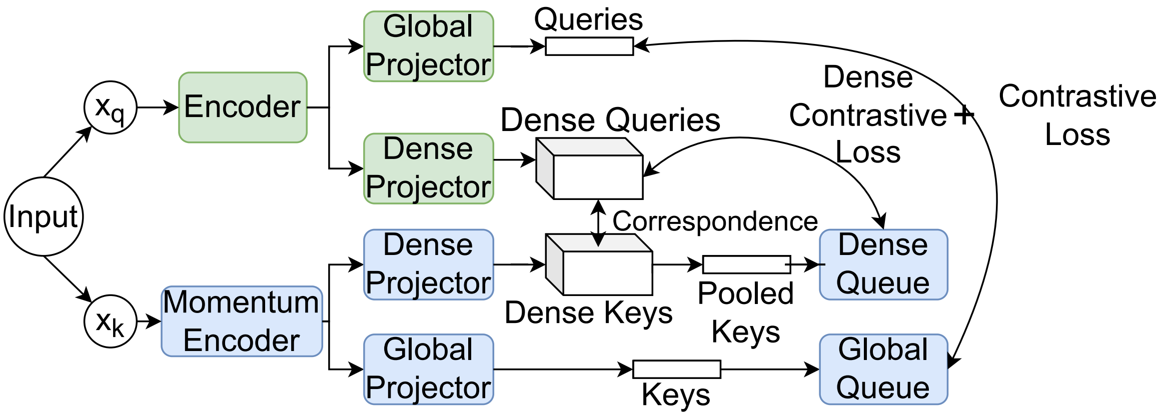

Figure S2: The pipeline for the dense contrastive learning framework. The encoder is a ResNet-50 network that outputs a dense feature map. The global projector includes a global average pooling layer and a 2-layer fully-connected network with a ReLU activation function in-between. The dense projector is a 2-layer fully convolutional network that applies 1x1 convolutions to feature maps. A global average pooling operation is applied to the dense keys prior to inclusion in the dense queue. All queries and keys have 128 dimensions.

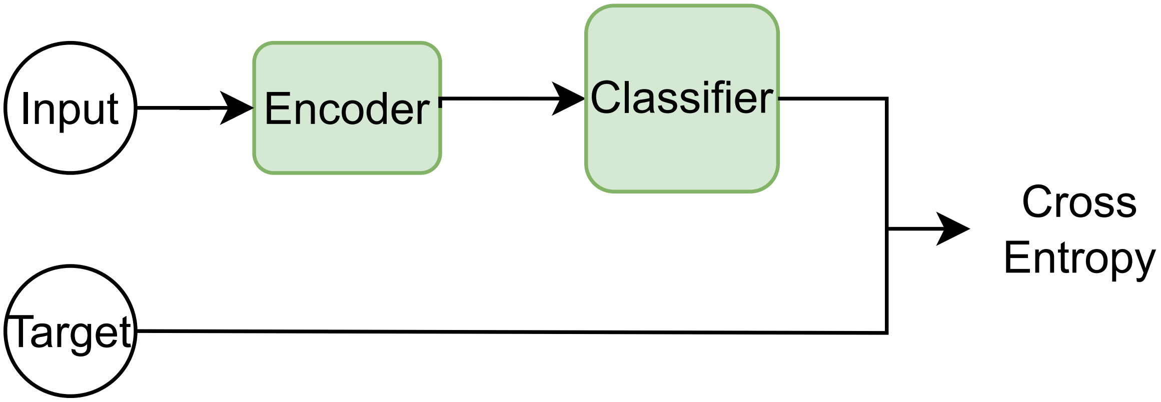

Figure S3: A schematic of the canonical supervised learning pipeline. The encoder is a ResNet-50 network that outputs a 2048-dimensional feature vector. The classifier is a singly fully-connected layer with as many neurons as the number of classes in the dataset.

B Additional Results

Figures

B.1 Optimal Transport Cost between Pre-training and Downstream Datasets

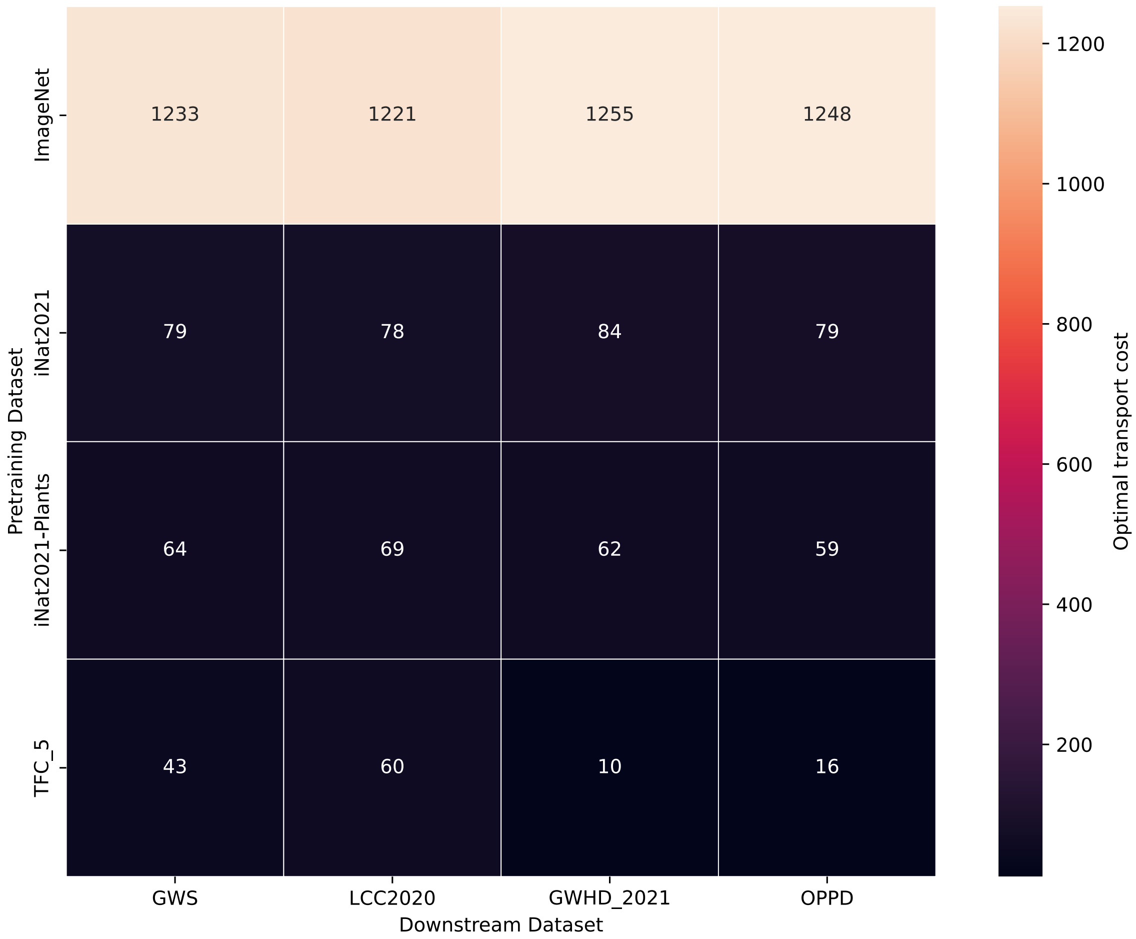

Figure S4: The optimal transport cost between the representations of the pre-training datasets and the downstream datasets. The optimal transport cost was computed[4]using 10,000 training samples for each of the pre-training datasets and all the training samples from each of the downstream datasets. The representations of the pre-training dataset were extracted from ResNet-50 encoders trained in a supervised way, while the representations for the downstream samples were extracted from models trained from random initialization. A higher number means it costs more to transform the probability distribution of one dataset into another.

[4]We used the following implementation: `https://github.com/kheyer/OTDD`

B.2 Domain Accuracy on the Wheat Head Detection Task

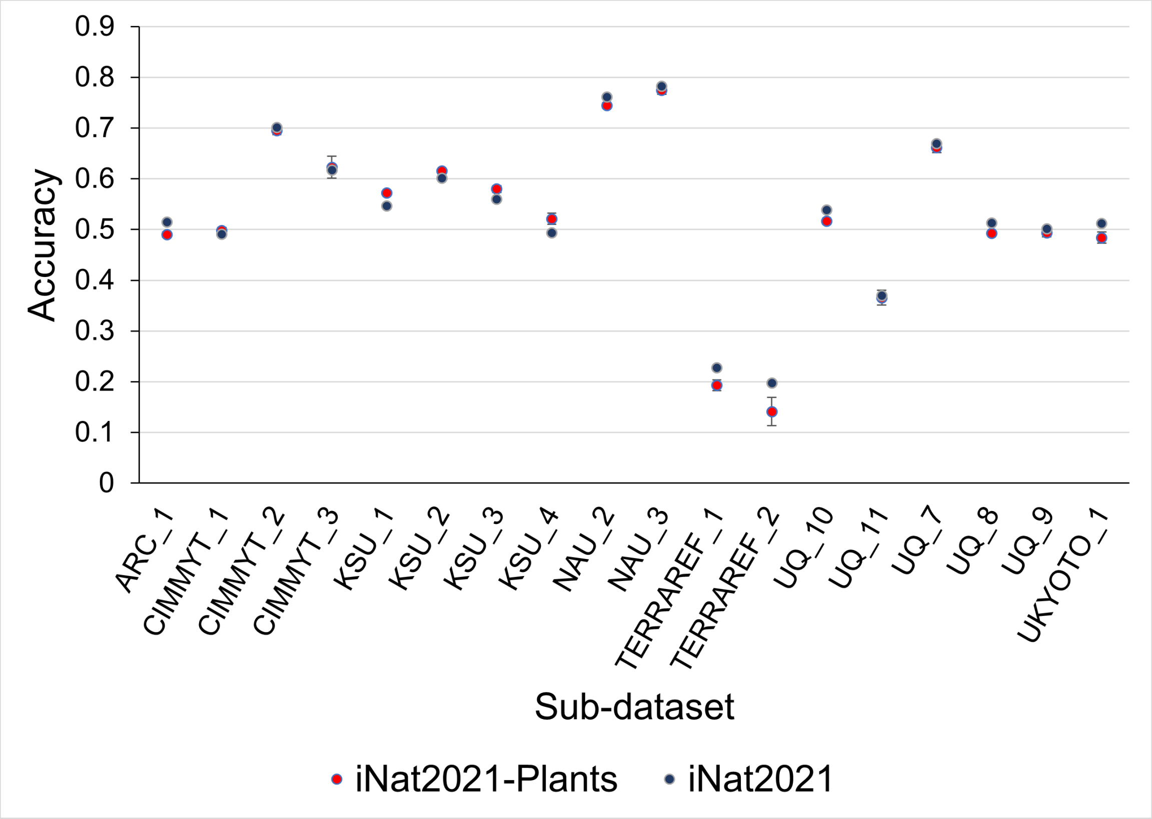

Figure S5: The accuracy for each domain/sub-dataset of the `GWHD_2021` dataset when fine-tuning models pre-trained with DenseCL on the iNat2021 and iNat2021-Plants datasets. The figure shows that the biggest differences in performance between the two pre-trained models are in the TERRAREF domains, leading the iNat2021 model to perform better on average.

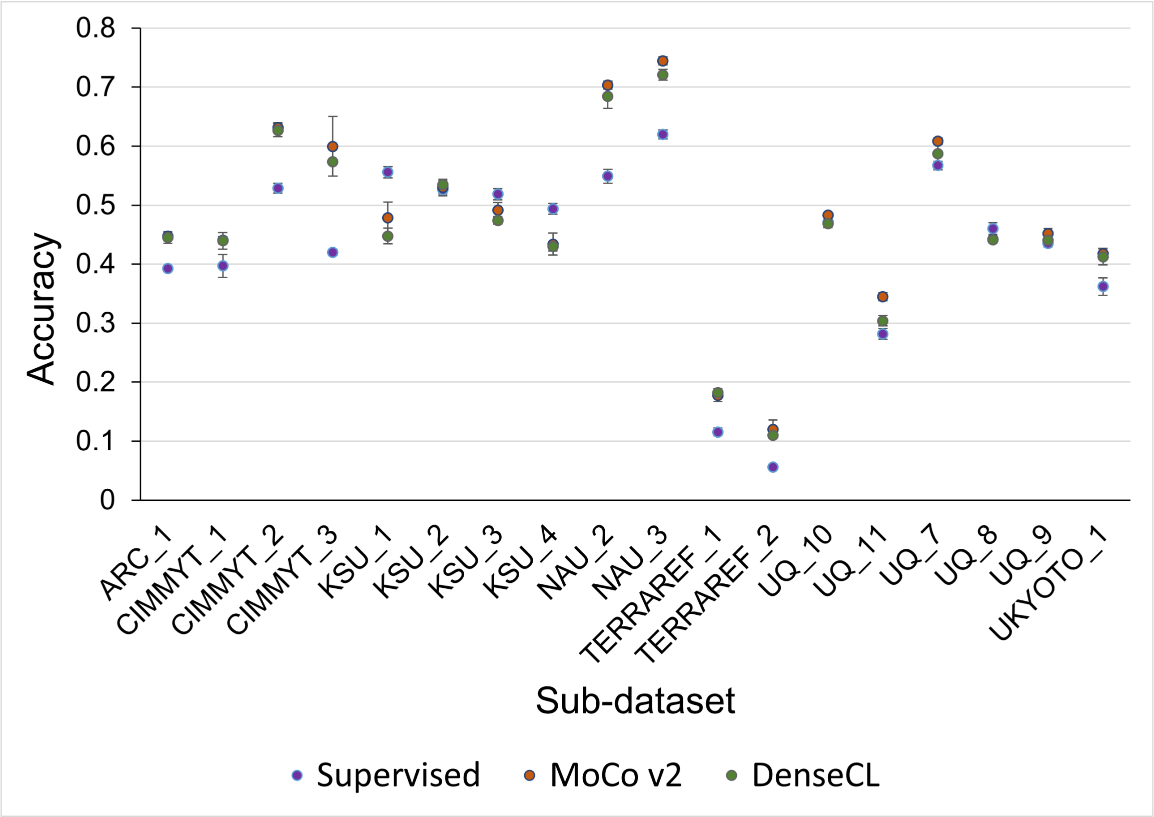

Figure S6: The accuracy for each domain/sub-dataset of the `GWHD_2021` dataset when fine-tuning models pre-trained on the `TFC_5` dataset with different methods. The figure shows that supervised pre-training underperforms across nearly all sub-datasets, while the performance of MoCo v2 and DenseCL are comparable across the sub-datasets.

B.3 Representation Similarity Analysis

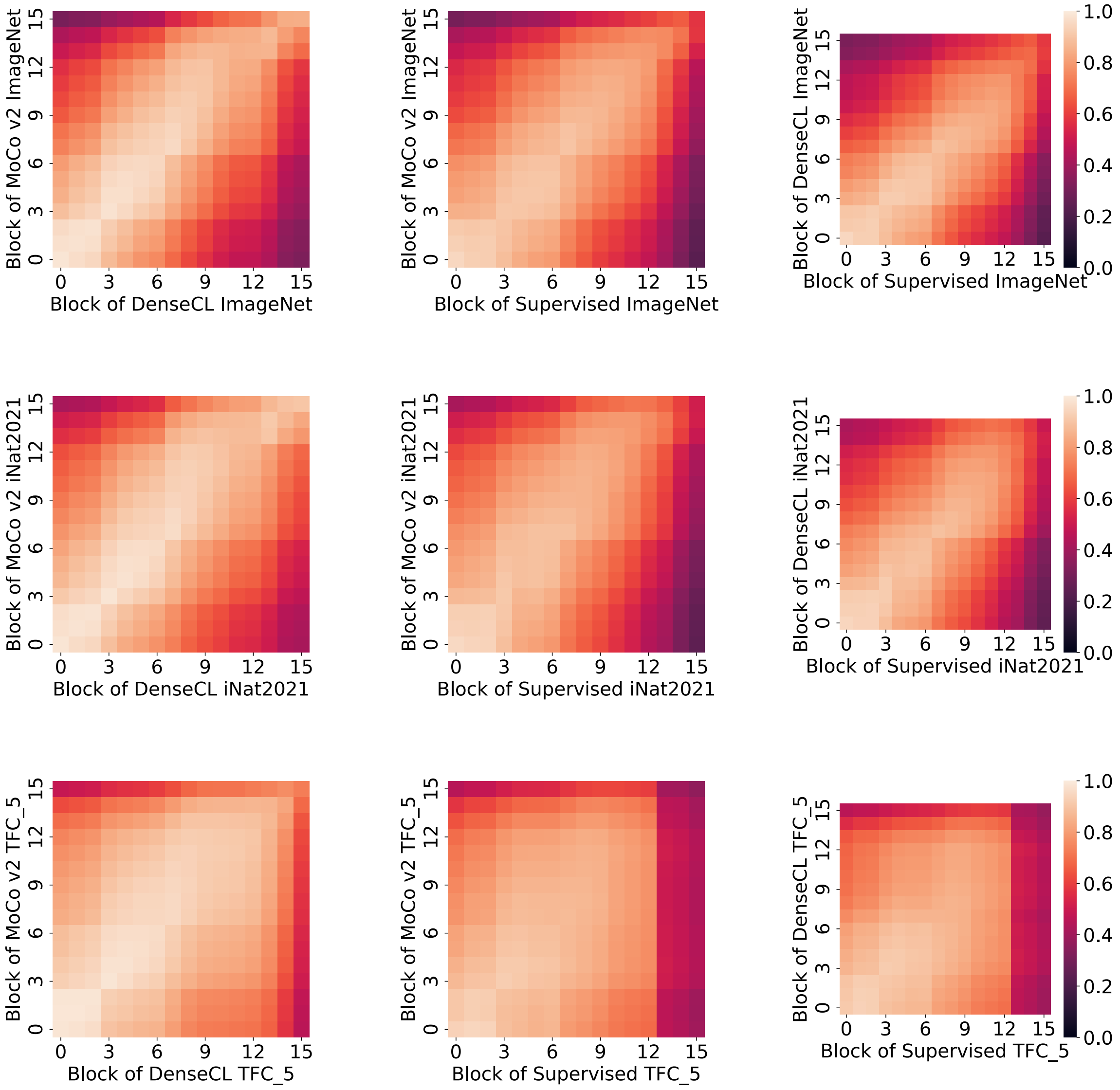

Figure S7: The Procrustes similarity metric between the output of ResNet-50 blocks from different pre-trained models.

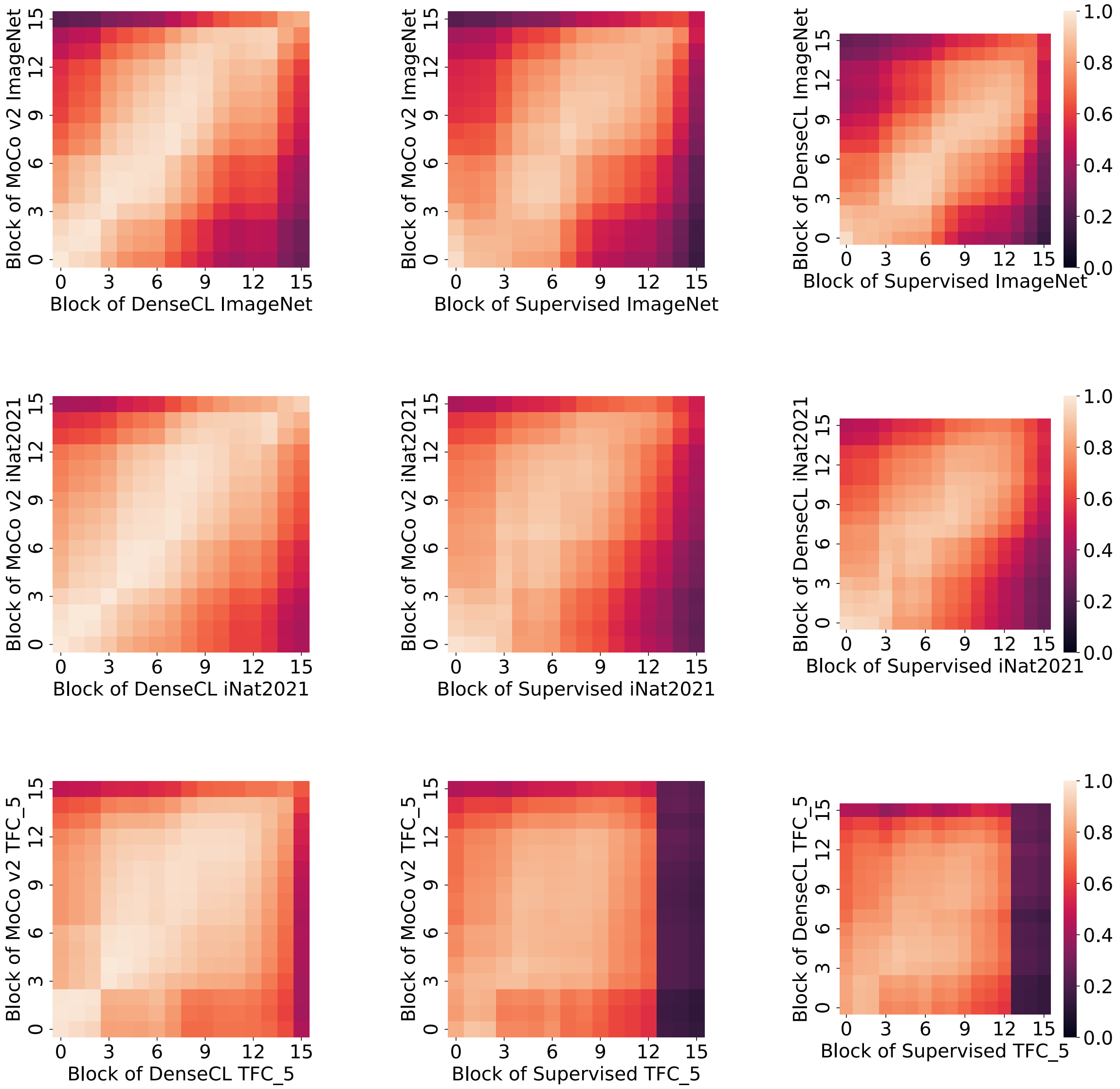

Figure S8: Linear CKA value between the output of ResNet-50 blocks from different pre-trained models.

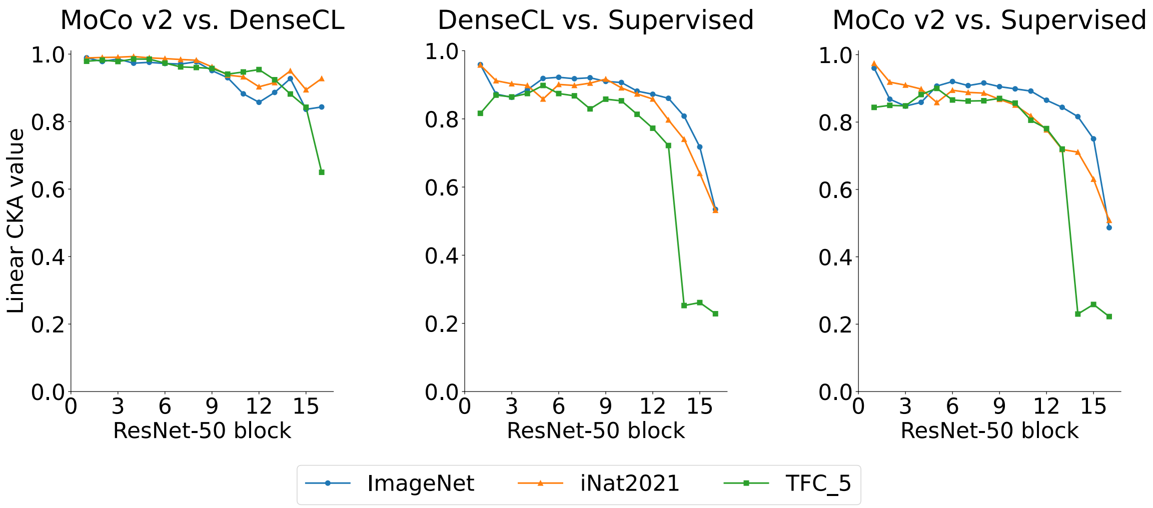

Figure S9: Linear CKA between the output of corresponding ResNet-50 blocks from different pre-trained models.

Tables

B.4 Effects of Data Redundancy on Downstream Tasks

Table S1: The table shows the performance when an encoder pre-trained on the subsets of the TFC dataset is finetuned on the downstream tasks. We report the mean (std) for the average domain accuracy (ADA) for the wheat head detection task, average precision (AP) for the plant instance detection task and mean absolute error (MAE) for the wheat spikelet and leaf counting tasks. The metric for the leaf counting task is computed on the validation and test sets.

Method	# of epochs	Wheat Head Detection		Plant Instance Detection	Wheat Spikelet Counting	Leaf Counting	
		ADA ↑	AP ↑	AP ↑	MAE ↓	MAE (val) ↓	MAE (test) ↓
Random Init	-	0.411 (0.003)	0.184 (0.003)	**0.423 (0.040)**	28.6 (4.2)	0.73	1.36
**Supervised**							
TFC_1	75	0.411 (0.005)	**0.245 (0.004)**	0.417 (0.040)	**19.9 (3.1)**	0.70	1.03
TFC_2	247	0.414 (0.002)	0.240 (0.002)	0.417 (0.040)	20.0 (2.2)	0.73	1.06
TFC_3	437	0.417 (0.005)	0.241 (0.003)	0.419 (0.041)	21.6 (2.1)	0.55	**0.82**
TFC_4	635	0.412 (0.004)	0.238 (0.004)	0.420 (0.040)	22.2 (4.0)	0.62	0.94
TFC_5	828	0.417 (0.004)	0.243 (0.003)	0.419 (0.039)	20.1 (2.9)	**0.53**	1.03
**MoCo v2**							
TFC_1	75	0.456 (0.002)	0.219 (0.004)	0.412 (0.038)	24.6 (6.2)	0.76	1.12
TFC_2	247	**0.472 (0.007)**	0.229 (0.003)	0.408 (0.036)	27.2 (4.9)	0.70	0.95
TFC_3	437	0.461 (0.003)	0.221 (0.005)	0.411 (0.038)	22.6 (3.2)	0.70	1.03
TFC_4	635	0.445 (0.003)	0.215 (0.006)	0.412 (0.038)	24.4 (7.0)	0.70	1.03
TFC_5	828	0.444 (0.008)	0.215 (0.004)	0.412 (0.039)	22.6 (4.2)	0.74	1.14
**DenseCL**							
TFC_1	75	0.463 (0.002)	0.230 (0.001)	0.415 (0.039)	22.0 (5.9)	0.64	0.98
TFC_2	247	0.460 (0.004)	0.228 (0.003)	0.412 (0.038)	26.1 (4.4)	0.70	1.11
TFC_3	437	0.465 (0.006)	0.231 (0.003)	0.412 (0.037)	22.5 (3.2)	0.68	1.18
TFC_4	635	0.454 (0.007)	0.223 (0.001)	0.411 (0.038)	22.1 (6.0)	0.70	1.15
TFC_5	828	0.466 (0.004)	0.233 (0.004)	0.414 (0.038)	27.4 (6.8)	0.66	1.11

B.5 Transfer Learning

Table S2: Wheat head detection performance with the GWHD_2021 dataset for different initialization schemes. We report the mean (std) for each metric. We show the overall best score for each metric in **bold letters** and underline the best score for each pre-training method.

Method	# of epochs	ADA	AP	AP_{50}	AP_{75}
Random init	-	0.411 (0.003)	0.184 (0.003)	0.473 (0.005)	0.108 (0.004)
**Supervised**					
ImageNet-1k [PyTorch]	90	0.496 (0.004)	0.241 (0.003)	0.534 (0.006)	0.179 (0.005)
ImageNet-1k [Ours]	200	0.527 (0.001)	0.268 (0.003)	0.601 (0.003)	0.200 (0.005)
iNat2021	95	0.554 (0.006)	0.276 (0.003)	0.607 (0.005)	0.210 (0.004)
iNat2021-Plants	223	**0.561 (0.006)**	**0.293 (0.004)**	**0.638 (0.005)**	**0.228 (0.006)**
TFC_5	1668	0.431 (0.002)	0.254 (0.004)	0.582 (0.005)	0.182 (0.003)
**MoCo v2**					
ImageNet-1k [25]	200	0.518 (0.003)	0.250 (0.003)	0.570 (0.005)	0.179 (0.003)
ImageNet-1k [Ours]	200	0.512 (0.005)	0.241 (0.002)	0.557 (0.005)	0.170 (0.002)
iNat2021	95	0.529 (0.003)	0.246 (0.002)	0.569 (0.005)	0.173 (0.003)
iNat2021-Plants	223	0.532 (0.002)	0.260 (0.004)	0.584 (0.005)	0.193 (0.005)
TFC_5	1668	0.472 (0.006)	0.225 (0.004)	0.523 (0.005)	0.158 (0.003)
**DenseCL**					
ImageNet-1k [32]	200	0.527 (0.003)	0.249 (0.003)	0.567 (0.004)	0.180 (0.005)
ImageNet-1k [Ours]	200	0.518 (0.004)	0.247 (0.003)	0.570 (0.005)	0.176 (0.004)
iNat2021	95	0.533 (0.002)	0.256 (0.002)	0.583 (0.004)	0.187 (0.003)
iNat2021-Plants	223	0.523 (0.003)	0.263 (0.000)	0.588 (0.002)	0.197 (0.002)
TFC_5	1668	0.462 (0.004)	0.230 (0.003)	0.526 (0.005)	0.167 (0.002)

Table S3: The transfer performance on the plant instance detection task with the OPPD dataset. We report the mean (std) for each metric. We show the overall best score for each metric in **bold letters** and underline the best score for each pre-training method.

Method	# of epochs	AP	AP_{50}	AP_{75}	AR_{1}	AR_{10}	AR_{100}
Random init	-	0.423 (0.040)	0.683 (0.062)	0.457 (0.046)	0.017 (0.003)	0.141 (0.026)	0.462 (0.044)
**Supervised**							
COCO [14]	-	0.370 (0.024)	0.655 (0.045)	0.373 (0.026)	0.012 (0.002)	**0.153 (0.022)**	0.441 (0.022)
ImageNet-1k [PyTorch]	90	0.424 (0.039)	0.677 (0.062)	0.459 (0.044)	0.018 (0.003)	0.143 (0.026)	0.461 (0.041)
ImageNet-1k [Ours]	200	0.429 (0.042)	0.689 (0.066)	0.465 (0.045)	0.018 (0.003)	0.144 (0.026)	0.467 (0.045)
iNat2021	95	0.431 (0.041)	0.689 (0.062)	0.469 (0.045)	0.018 (0.004)	0.144 (0.027)	0.466 (0.045)
iNat2021-Plants	223	**0.438 (0.041)**	**0.695 (0.062)**	**0.471 (0.046)**	**0.018 (0.003)**	0.145 (0.027)	**0.474 (0.045)**
TFC_5	1668	0.425 (0.039)	0.687 (0.063)	0.459 (0.042)	0.018 (0.003)	0.142 (0.026)	0.464 (0.043)
**MoCo v2**							
ImageNet-1k [25]	200	0.401 (0.035)	0.661 (0.058)	0.432 (0.039)	0.018 (0.003)	0.140 (0.025)	0.441 (0.038)
ImageNet-1k [Ours]	200	0.414 (0.038)	0.671 (0.059)	0.445 (0.041)	0.018 (0.003)	0.142 (0.026)	0.450 (0.040)
iNat2021	95	0.417 (0.037)	0.672 (0.059)	0.449 (0.042)	0.018 (0.004)	0.142 (0.026)	0.452 (0.040)
iNat2021-Plants	223	0.423 (0.039)	0.681 (0.059)	0.455 (0.043)	0.018 (0.004)	0.143 (0.026)	0.458 (0.041)
TFC_5	1668	0.412 (0.038)	0.670 (0.058)	0.428 (0.041)	0.017 (0.003)	0.140 (0.026)	0.449 (0.040)
**DenseCL**							
ImageNet-1k [32]	200	0.424 (0.040)	0.678 (0.064)	0.458 (0.045)	0.018 (0.003)	0.143 (0.027)	0.460 (0.043)
ImageNet-1k [Ours]	200	0.412 (0.038)	0.670 (0.058)	0.442 (0.043)	0.018 (0.004)	0.141 (0.026)	0.450 (0.041)
iNat2021	95	0.420 (0.038)	0.678 (0.060)	0.450 (0.044)	0.018 (0.003)	0.143 (0.026)	0.454 (0.041)
iNat2021-Plants	223	0.424 (0.040)	0.682 (0.059)	0.456 (0.045)	0.018 (0.004)	0.143 (0.026)	0.458 (0.042)
TFC_5	1668	0.412 (0.038)	0.670 (0.058)	0.441 (0.043)	0.018 (0.003)	0.140 (0.026)	0.450 (0.041)
